# Landscape homogenization due to agricultural intensification disrupts the relationship between reproductive success and main prey abundance in an avian predator

**DOI:** 10.1186/s12983-019-0331-z

**Published:** 2019-08-06

**Authors:** Petra Sumasgutner, Julien Terraube, Aurélie Coulon, Alexandre Villers, Nayden Chakarov, Luise Kruckenhauser, Erkki Korpimäki

**Affiliations:** 1 Department of Biology, Section of Ecology, University of Turku, Turku, Finland; 2 Department of Integrative Zoology, University of Vienna, Vienna, Austria; 3 FitzPatrick Institute of African Ornithology, DST-NRF Centre of Excellence, University of Cape Town, Cape Town, South Africa; 4 Global Change and Conservation Lab. Faculty of Biological and Environmental Sciences, University of Helsinki, Helsinki, Finland; 5 CEFE, CNRS, Univ Montpellier, Univ Paul Valéry Montpellier 3, Montpellier, France; 6 Centre d’Ecologie et des Sciences de la Conservation (CESCO), Muséum national d’Histoire naturelle, Centre National de la Recherche Scientifique, Sorbonne Université, Paris, France; 7 ONCFS, Unité Avifaune Migratrice, Station de Chizé, 405 route de Prissé-la-Charrière, 79360 Villiers-en-Bois, France; 8 Department of Biology, Molecular Ecology and Evolution Lab, Lund University, Lund, Sweden; 9 Department of Animal Behaviour, Bielefeld University, Bielefeld, Germany; 10 Museum of Natural History Vienna, Central Research Laboratories, Vienna, Austria

**Keywords:** Agro-ecosystems, Biodiversity conservation, Boreal landscapes, Eurasian kestrel, Global change, Heterozygosity–fitness correlations, Individual quality, Predator-prey interactions

## Abstract

**Background:**

Selecting high-quality habitat and the optimal time to reproduce can increase individual fitness and is a strong evolutionary factor shaping animal populations. However, few studies have investigated the interplay between land cover heterogeneity, limitation in food resources, individual quality and spatial variation in fitness parameters. Here, we explore how individuals of different quality respond to possible mismatches between a cue for prey availability (land cover heterogeneity) and the actual fluctuating prey abundance.

**Results:**

We analyse timing of breeding and reproductive success in a migratory population of Eurasian kestrels (*Falco tinnunculus*) breeding in nest-boxes, over a full three-year abundance cycle of main prey (voles), and consider several components of individual quality, including body condition, blood parasite infection, and genetic diversity (*n* = 448 adults) that act on different time scales. Older individuals, and kestrel parents in higher body condition started egg-laying earlier than younger birds and those in lower body condition. Additionally, egg-laying was initiated earlier during the increase and decrease phases (2011 and 2012) than during the low phase of the vole cycle (2013). Nestling survival (ratio of eggs that fledged successfully) was higher in early nests and in heterogeneous landscapes (i.e., mosaic of different habitat types), which was evident during the increase and decrease phases of the vole cycle, but not during the low vole year.

**Conclusions:**

We found a strong positive effect of landscape heterogeneity on nestling survival, but only when voles were relatively abundant, whereas a difference in the timing of breeding related to territory landscape heterogeneity was not evident. Therefore, landscape heterogeneity appeared as the main driver of high reproductive performance under favourable food conditions. Our results show that landscape homogenization linked to agricultural intensification disrupts the expected positive effect of vole abundance on reproductive success of kestrels.

**Electronic supplementary material:**

The online version of this article (10.1186/s12983-019-0331-z) contains supplementary material, which is available to authorized users.

## Background

Selecting the right time and place to reproduce is an essential decision for animals. It will affect their reproductive output and long-term survival, and as a result what we refer to as individual fitness [1, 2]. For territorial birds, the fittest individuals are expected to preferentially occupy the higher-quality sites, while less competitive individuals occupy poorer sites (‘Ideal Dominance Distribution model’, [3–5]). In migratory species, earlier-arriving individuals are usually of higher individual quality (for instance older and/or in better body condition) than later-arriving ones and settle on progressively lower-quality territories (‘sequential settlement’, see [5]). Early arrival may therefore be advantageous in terms of reproductive performance (reviewed in [6]).

Under habitual environmental conditions, one would expect territories occupied first to show highest breeding success as a combination of higher quality individuals occupying higher quality territories. But rapid global changes, such as climate change and land-use changes, have severely altered the relationship between species and their environment [7, 8]. Large-scale changes in land-cover can have detrimental effects on species either through habitat loss and fragmentation [9, 10], or in a more devious manner, by uncoupling cues used to select a suitable habitat, and the true value of this habitat [11]. This type of uncoupling has been shown in many ground-nesting birds that persist to breed in intensive farmlands because they are lured by cues that used to be appropriate in pristine open habitats [2]. For example, Northern Wheatears (*Oenanthe oenanthe*) use field layer height as a cue for quality, but height also correlates with the temporal proximity to harvesting. Thus, intensive farmlands are associated with poor reproductive success due to agricultural activities destroying clutches or broods, or reducing adult survival [12, 13]. Identifying the existence of cue mismatches and unravelling the mechanisms behind them is necessary to fully understand and predict the impacts of land-use changes on population dynamics.

One main challenge in unravelling mechanisms behind potential mismatches between perceived quality and realised fitness, is the biologically valid definition and quantification of habitat quality and individual quality. Studies that combine both measures are still rare (but see for example [5, 14, 15]). Additionally, such individual quality measurements might operate on varying time scales. For example, body condition may vary in time and as a consequence captures only a relatively short time period. Blood parasite infection would act on a medium time frame which incorporates the exposure and susceptibility to haemosporidian parasites. Gene diversity expressed by individual genetic heterozygosity would be constant over time. In more detail, body condition is a measure of fat content or nutrient reserves in relation to body size. It can indicate periods of nutritional stress [16, 17] and might vary between and within years (i.e., over the course of a breeding season; [18]). Habitat composition might also determine the exposure to vectors of blood parasites [19, 20]. At the same time, if a bird’s immune system is compromised due to nutritional stress [21] or breeding effort [22], the infection risk (probability of infection, estimated from the presence or absence of haemoparasites at the individual level) might increase. Both, exposure and susceptibility to vector-borne parasites will thus reflect the quality of a birds’ settlement decision but also the individual quality of the breeding adult that might again vary over time. Infection status might further shape reproductive investment strategies and decrease reproductive success, since individual’s defence against parasites is costly [23]. Lastly, individual genetic heterozygosity (i.e., proportion of genetic loci bearing two different alleles, reflecting the genetic diversity of an individual) is known to affect fitness-related traits (see heterozygosity–fitness correlations; e.g. [24–26]). For example, heterozygosity (estimated by microsatellites) in females can be positively associated with clutch and egg sizes [27]. Heterozygous mates often provide larger food items [28] in higher feeding rates [29]. Another commonly used individual quality measurement is the age of the breeding adults, that relates to breeding experience, and is well known to influence both the timing of breeding and breeding success (as for example documented in our study system, e.g.: [30–32]).

European farming policies have led to a collapse in farmland bird populations [33]. The Eurasian kestrel (*Falco tinnunculus,* hereafter kestrel) is no exception to this general negative trend [34, 35]. In Finland, kestrel populations collapsed because of pesticides in late 1950s and 1960s, and have then been increasing during 1980s to 2000s thanks to the provisioning of suitable nest-boxes in certain areas [36]. In Northern Europe, kestrels are long-distance migrants that over-winter in Southern Europe, North Africa and the Sahel region [37, 38]. They are capable of breeding in various habitats as long as open landscape for hunting is available. We hypothesize that early migrants prefer to settle in open habitats, generated by intensive agricultural practices, because in boreal areas, heterogeneous habitats with forested patches are expected to retain snow for a longer period than open farmlands. Indeed many studies have investigated the effects of vegetation types on snow accumulation and melting processes and have shown that snow melt rates are slower in forested landscapes ([39–41]; and see [42] for a thorough review of the empirical evidence in the literature). The early disappearance of snow in homogeneous agricultural fields could hence be an anthropogenically induced cue for habitat quality because voles, kestrels’ main prey [38, 43], are more easily detectable in snow-free patches during the settlement phase [44]. However, this cue can be biased on some years, depending on the phase of the vole cycle which follows a high-amplitude 3-year population cycle in western Finland [45]. In low vole abundance years, it is likely that homogeneous agricultural habitats are of lower quality for kestrels than more heterogeneous habitats, where more alternative prey species (birds, shrews, lizards and insects) can be found [46, 47]. Additionally, organic farming (following the agri-environment-climate schemes of the European Union, including reducing land-use intensity and maintaining or introducing biodiversity-rich habitats) and the amount of non-field grassland are positively correlated to total bird abundance across Finland [48]. Kestrels are indeed known to hunt in forests and clear-cut areas during poor vole years [49]. Therefore, this species offers a unique opportunity to disentangle the underlying mechanisms of how variations in landscape heterogeneity and main prey abundance may affect the reproductive success of an avian predator depending on individual quality.

Here, we explore how individuals of different quality respond to possible mismatches between a cue for prey availability (land cover heterogeneity) and the actual fluctuating prey abundance. We used laying date as a proxy of time of settlement, and nestling survival (the ratio of laid eggs that successfully fledged) as a proxy for breeding investment versus breeding performance. We expected high-quality individuals to arrive earlier on breeding grounds and to settle in more homogeneous agricultural landscapes where snow melts earlier and where prey might be more accessible. We predicted that (i) egg-laying initiates earlier in homogeneous agricultural fields; and, (ii) high-quality individuals start egg-laying earlier in more homogeneous landscapes, and low-quality individuals start egg-laying later in more heterogeneous landscapes. We further predicted these patterns to be independent of the vole cycle: (iii) high-quality individuals manage to have high nestling survival in homogeneous landscapes when voles are abundant, but they are ‘trapped’ in these territories with few alternative prey during low vole abundance years, which will be seen in low nestling survival. In turn (iv) low-quality individuals have higher nestling survival than high-quality individuals in heterogeneous landscapes during low vole abundance years only. Finally, we predicted (v) that short-term individual quality measures and breeding experience (parental age) have a stronger influence under such fluctuating food conditions than long-term individual quality measurements.

## Methods

### Study area

**Fig. 1:**
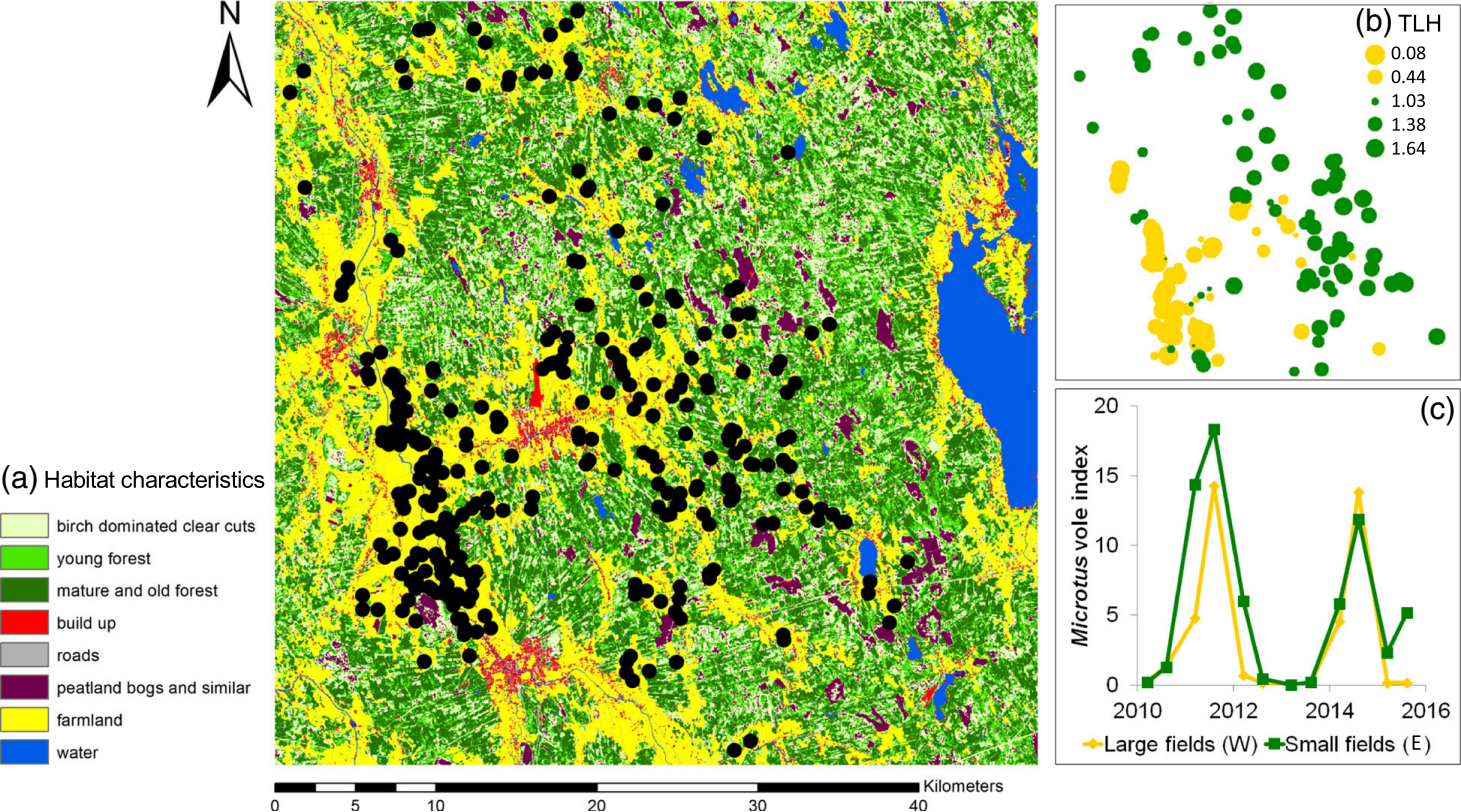
**a** Study area in the Kauhava region, Western Finland, consisting of a mix of mainly two contrasting habitats: homogeneous open habitat in the West and heterogeneous habitat in the East; black dots indicate kestrel nest-boxes. **b** Territory land cover heterogeneity (TLCH) for kestrel territories in the study area; represented as min (TLCH = 0.03), 1st quartile (TLCH = 0.18), median (TLCH = 0.49), 3rd quartile (TLCH = 0.68) and max (TLCH = 0.79) value (TLH > median shown in green, TLH < median shown in yellow), whereby smaller THL scores indicate homogenous landscapes, and higher TLH scores indicate heterogeneous landscapes. **c** Periodic 3-year vole cycle covering the study period 2011–2013 and showing the increase (2011), decrease (2012) and low phase (2013), based on snap-trapping data (no. of *Microtus* voles trapped per 100 trap-night) in spring (May) and autumn (Sep), in two sampling plots in large fields (homogenous landscapes in the West) and small fields (heterogenous landscapes in the East) respectively, of the study area

The study area is situated in the Kauhava and Lapua region, western Finland (62°59′-63°10′N, 22°50′- 23°20′E, see [50]). It consists of a mix of two contrasted habitats: the first is a homogeneous open habitat (mostly in the West, > 70% agricultural fields) and the second is a heterogeneous habitat (mostly in the East, 25–30% agricultural fields interspersed with exploited coniferous forests and clear-cuts; Fig. 1a). A majority (60–70%) of agricultural fields of the study area are sown every spring with mostly oats and barley, and correspondingly a minority (30–40%) of agricultural fields are permanently producing grass for silage and hay. Cereal fields are usually ploughed already in autumn. Over the course of the 3-years study period, we did not see any changes in temperature, precipitation or snow cover. For an overview of long-term weather data, see Fig. 3.5. in [51].

#### Territory characteristics

Yearly land cover maps were produced by using Landsat images in a resolution of 30x30m (see [52] for details on how the maps were generated). Landscape heterogeneity was defined as the diversity of land covers in a kestrel territory and was calculated with Simpson’s Index (see [53]) in the ‘vegan’ package [54], by including the number of land cover categories present and the percentage area of the following types: built-ups and roads, peatland bogs, agricultural fields, water, clear-cuts, young forest, mature and old-growth forest (more details can be found in Additional file 1: S1 and in Appendix S2 in [52]). Forests were classified according to their wood volume, following the Finnish Forest Resources Institute classification: clear cuts: < 52 m^3^/ha, young forests 52–101 m^3^/ha, mature forests 102–151 m^3^/ha and old forests > 152 m^3^/ha. We used the average inter-nest distance between occupied boxes as a proxy for the diameter of kestrel territories (i.e., an average of all nest distances to their closest neighbour) and hence as buffer radius to characterise landscape heterogeneity in each territory. The vole cycle highly influences the breeding density of kestrels [50], to account for this varying density, we varied the buffer scale accordingly between years: r_(2011)_ = 650 m (132.7 ha); r_(2012)_ = 694 m (151.3 ha), r_(2013)_ = 843 m (223.3 ha). We used the Simpson’s Index to quantify Territory Land Cover Heterogeneity (see [55] for a similar method of characterising raptor territory composition), hereafter called ‘TLCH’, whereby lower values represent more homogeneous landscapes, and higher values more heterogeneous landscapes (Fig. 1b). Homogeneous landscapes mainly consist of large agricultural fields (correlation between TLCH and farmland areas plotted in Additional file 1: S2). Additionally, we measured the distance between the nest box and the closest forest edge to account for potential higher predation risk imposed by larger forest predators such as Northern goshawks (*Accipiter gentilis*) and Pine martens (*Martes martes*). These two species often depredate kestrel nests and adults [56]. Real and perceived predation risk can affect breeding habitat selection, onset of breeding, and clutch size in a range of species (e.g. [57–60]). In addition, it has previously been shown that kestrel nest boxes located close to forest edges have a lower occupancy rate than nest boxes located away from forest edges [61], perhaps as a response to the perceived risk of predation near forests. Finally, we controlled for potential density dependent effects on laying date and breeding success by including the nearest neighbour distances (NND, in meters, log transformed) between occupied nest boxes in the analyses. We chose NND because studies exploring different measurements found the distance to nearest neighbours to be the best predictor for density dependence (e.g., [62]).

#### Vole density index

The main prey items of kestrels in our study site are voles of the genera *Microtus* (the field vole *Microtus agrestis* and the sibling vole, also called southern vole, *Microtus rossiaemeridionalis*) and *Myodes* (the bank vole *Myodes glareolus*, [43]). Their abundance is highly determined by the phase of the high-amplitude 3-year vole cycle [45, 63]. No long-term temporal trend in abundance of *Microtus* and bank voles could be detected in our study area and regular three-year cyclic fluctuations of these voles have been evident from 1980s onwards up to 2010s (Additional file 1: Figure S5; [64]). The phase of the vole cycle largely determines breeding density and performance of kestrels: breeding density is higher, egg-laying starts earlier, clutch sizes are larger, and breeding success is higher in the increase and decrease phases than in the low phase of the 3-year vole cycle [50, 65]. We covered a complete cycle between 2011 and 2013 and estimated vole abundance indices by bi-annual trapping (Fig. 1c). Rodent abundance in the study area is spatially synchronous (over 70–500 km, see [66–69]) and does not show differences between the two types of habitats, which is also known from other boreal study systems that undergo strong population cycles in vole abundance [70]. Snap traps were set up in mid-September (autumn) and early May (spring) in two sites 14 km apart in the study area. In each of these two sites, four plots were sampled in the main habitat types including cultivated fields, abandoned fields, spruce forests and pine forests (see [45] for details). Between 50 and 60 baited Finnish metal mouse snap traps were set at 10-m intervals in vole runways on each plot and were checked daily for 3 consecutive days. Thus, the area of a sample plot ranged from 0.5 to 0.6 ha. We pooled the trapping results for *Microtus* voles from four-night trapping periods and standardised them to the number of animals caught per 100 trap nights in each habitat type. These data are thereafter referred to as ‘vole index’ in ‘spring’ and ‘autumn’. The vole cycle in the study area has been documented with this method since 1973 (see long-term *Microtus* vole index for the study area in Additional file 1: S3).

#### Kestrel sampling

In the study area, kestrels have been breeding in nest-boxes fastened on the gables of barns since the early 1980’s. The proportion of tree breeders is below 10% (EK, pers. obs.). Between 2011 and 2013, a total number of 358 (2013), 363 (2012) and 374 (2011) kestrel nest-boxes were monitored in the study area. The study population is known for its high annual turn-over rate of 82%, together with a high divorce rate (on average 90% females and 68% males are new in our study area, pooled data from 1985 to 2010; see [71]). From the beginning of the breeding season (late April to early May), nest-boxes were inspected 3 to 4 times per breeding season to record the occupancy, egg-laying date, clutch size and number of fledged young of all active nests while minimizing nest disturbance. Kestrels start incubation after the second egg is laid, therefore the lay date can be estimated by subtracting 30 days from the estimated date of hatching [38], which can be determined on the basis of wing lengths of nestlings for successful nests [72]. For unsuccessful nests, we used egg-floating, a method well-established in waders [73–75] that was modified for kestrels to estimate approximate day of hatching. Parents were trapped at the nests by using a swing-door trap attached on the entrance hole of the nest box when chicks were 2–3 weeks old (see [71] for details on morphometric measurements and blood sampling). They were ringed and aged according to [76, 77]. We obtained morphometric measurements for the body condition index and blood samples for *Haemoproteus* sp. infection and genotyping of 448 individuals (n_(2011)_ = 139, n_(2012)_ = 163 and n_(2013)_ = 151 adults). In our 3-year data set, we did not have any repeated measurements of breeding adults, which is due to long breeding dispersal distances and the high turnover rate of breeding individuals in the study population [71].

#### Individual quality

We quantified indices for individual quality that act on different time scales: i) body condition; ii) blood parasite infection, iii) genetic diversity; and iv) age as a proxy for breeding experience.

I) The residuals of the regression of body mass on wing length (both log transformed), sex and the time (in days) between the capture date and laying date were used as an index of body condition (abbrev. ‘bc’; [78]), in order to account for the size dimorphism of the species (females are larger than males) and the decrease in body mass of adults throughout the breeding season [18, 79]. Lower values reflect individuals in lower body condition and vice versa; see [80] for a similar approach.

II) DNA extractions of collected blood samples were done in the Center of Evolutionary Applications, University of Turku, and were subsequently genetically screened for blood parasites of the genera *Haemoproteus*, *Plasmodium* and *Leucocytozoon* at the Molecular Ecology and Evolution Lab, Lund University, Sweden. The exact protocol based on a nested PCR can be found in Additional file 1: S4. Only *Haemoproteus nisi* was detected with a sufficient prevalence and was included as the blood parasite infection risk variable (presence/absence in an individual adult). Blood parasite prevalence does not change throughout an individuals’ life once an infection occurred, as opposed to infection intensity, that varies over time [81].

III) The laboratory work for individual genetic heterozygosity was done in the Central Laboratories of the Natural History Museum Vienna, Austria and was based on 22 different microsatellites established for *Falco peregrinus* [82] and *F. naumanni* [83, 84]. Details on the primers used and the multiplex PCR protocol can be found in Additional file 1: S5, the exact procedure to i) determine final allele sizes; ii) identify outliers; iii) test for potential scoring errors, allelic dropout and null alleles; iv) departures from Hardy-Weinberg equilibrium; and, v) genotypic linkage disequilibrium can be found in Additional file 1: S6. After this quality assessment, the remaining 17 microsatellites were used to estimate five commonly used measures of individual multilocus heterozygosity using the GENHET R function [85]. However, because of the high inter-correlation among these measures (Spearman correlations: |r| > 0.95, *P* < 0.001), we chose only one, the standardized heterozygosity [25]. This is a widely used and effective measure of genetic diversity, computed as the proportion of heterozygous loci for a given individual divided by the average of the population-level mean heterozygosity for those same loci ([25]; see [86, 87] for a similar approach), and is used hereafter as ‘Hs_exp’, whereby lower values reflect lower genetic heterozygosity.

IV) The age estimation in kestrels is only possible between first year breeders and older individuals (see [76, 77]). Age was hence included as a two-level factor variable in our analyses. In raptors, young breeders usually start egg-laying later (e.g., [30, 50]) and have lower breeding success (e.g., [31, 32]). As mentioned before, the high turn-over and divorce rate in our study population does not leave us with many ringed adults that would allow a more exact age estimation.

#### Statistical analyses

To analyse how individual quality, territory land cover heterogeneity and vole abundance influence the timing of breeding (estimated as lay date; to test predictions i and ii) and breeding performance (estimated as nestling survival, to test predictions iii and iv) we used an information theoretic approach and model averaging with the R packages ‘lme4’ [88], “MASS” and ‘MuMIn’ [89]; see [90, 91] for details on multi-model inference. Model averaging was the appropriate approach, as no single model was strongly supported for either response variable [92]. The vole indices in spring and autumn were correlated (*r* = 0.50), thus we initially fitted competing models and decided to use the vole abundance in spring as the predictor variable throughout because of higher explanatory capacity and because the index in spring, when kestrels arrive on territory, was more biologically meaningful from the point of view of kestrels making settlement decisions. The vole index was fitted as an ordered factor in the statistical analyses. Results in the model output are thus displayed for the linear or quadratic relationship between the 3 study years/vole abundances.

Lay date (Julian day of clutch initiation, following a normal distribution) was analysed using LMMs with a Gaussian error distribution and identity link function and the following fixed-effects: individual quality (either body condition index, *Haemoproteus* sp. infection risk or individual genetic heterozygosity [Hs_exp] as models tend to fail to converge if all three uncorrelated variables were included at once), age (factor with two levels: first year breeder or older), TLCH and the vole index in spring, together with the interaction terms between individual quality*TLCH, individual quality*vole index, and vole index*TLCH. Finally, the models included the distance to the closest forest edge (dist) and the nearest neighbour distances (NND) as additional fixed effects. ‘Nest box ID’ was included as random factor. In the lay date analyses all breeding records (successful and failed nests) were included.

Nestling survival was estimated as the ratio of laid eggs that successfully fledged. Some eggs that did not result in fledglings may have been infertile, which may vary according to habitat composition (e.g., [93]), thus our variable was a combination of egg-hatch ratio and hatch-fledged ratio, but hatching numbers were not known for all broods, explaining why we could not separate between the two contributing factors. It was analysed using GLMMs with a binomial denominator. We used the same fixed-effects as for the timing of breeding [individual quality (either body condition index, *Haemoproteus* sp. infection risk or Hs_exp), age, TLCH, vole index, individual quality*TLCH, individual quality*vole index, vole index*TLCH, dist and NND], with the relative lay date (centred to the mean of the study year) as an additional predictor variable because of the known decline in clutch size with later laying date in kestrels [50]. ‘Nest box ID’ was again included as a random factor. In the nestling survival analyses, only successful nests that fledged at least one young were included. We found 8 complete nest failures during the study period, that happened after adult trapping (when nestlings are 2–3 weeks old) and might have been due to predation.

All fixed effect covariates were tested beforehand for correlations; and with the exception of the two vole indices, no strong correlations were found (and all predictors with rho< 0.4 were maintained as covariates). Predictor variables contained no missing values, ensuring accurate model comparisons throughout the selection and averaging process [94]. A global model was fitted with any strongly correlated explanatory variables; all quantitative variables were scaled and centered, ensuring that effect sizes were on a comparable scale [95]. We generated a candidate list using all possible combinations of the predictors outlined above. Additionally, the appropriate null models (i.e., random factors ‘nest box ID’ only, and yearly variation ‘vole index’ only), were considered in the candidate list (but never featured into any of the top models). See Additional file 1: S7 for the complete candidate lists. (Note that the vole index and ‘year’ were fully confounded (*r* = 1.0), which is why study year was not further considered in the candidate models, and could also not be considered as a random term because 3 levels are not sufficient [96]. But because of the full correlation between ‘year’ and vole abundance, the yearly variation is fully accounted for throughout. Each candidate model was compared to one another using Akaike Information Criterion values, corrected for small sample size (AICc) in the package ‘AICcmodavg’ [97]. Akaike weights (ω_i_) were calculated to assess the relative likelihood for each model considered [98]; thus, ω_i_ reflect the model probability given the full model list rather than only those below a given threshold of ΔAICc. All models with ΔAIC_c_ < 4.0 were extracted and consequently used for model averaging [99]. We report the direction of parameter estimates and their magnitudes (effect sizes), unconditional SEs and CIs (95% confidence intervals) from model averaged coefficients, and the variable’s relative importance (RVI; i.e., model probability for each explanatory variable tested; [98, 100]. Unconditional SEs incorporate model selection uncertainty, as opposed to standard SEs which only consider sampling variance [90, 91]. We used CIs to assess the magnitude of the effect and conclude that the estimate is different from zero (i.e., there is a significant effect) when the CIs exclude zero. Posthoc comparisons between factor variables in interaction terms were performed using the package ‘emmeans’ [101]. All statistical analyses were performed with the software R version 3.4.4 [102] unless stated otherwise. The confidence intervals were set at 95% (corresponding to a significance level of *P* = 0.05) for all tests conducted.

For both model selection processes, we visually inspected residual distribution to assess model fit and tested for potential spatial autocorrelation in all response variables by using Moran’s I (“ape” package, [103]) and visual inspection of spatial plots and variograms of residuals (“gstat” package, [104]). We found no indication for spatial autocorrelation throughout (P ∈ [0.36; 0.82]). We present spatial plots for the individual quality indices i) body condition (Gaussian distribution); (ii) *Haemoproteus* infection risk (binomial distribution); and, (iii) Hs_exp (Gaussian distribution) for both sexes in Additional file 1: S8.

## Results

The year 2011 was an increase phase of the vole cycle (*Microtus* vole index autumn 2010 = 1.29, spring 2011 = 9.58, autumn 2011 = 16.2) with a following decrease phase (spring 2012 = 3.36, autumn 2012 = 0.28). The next year (2013) was a low vole year (spring 2013 = 0 and autumn 2013 = 0.17 *Microtus* voles trapped per 100 trap nights). Nest box occupancy heavily depended on the phase of the vole cycle. In 2011, the increase phase of the vole cycle, 222 nest boxes were occupied (59%), 219 of which successfully fledged young (98%). In 2012, the decrease phase of the vole cycle, 199 nest boxes were occupied (55%), all of which were successful (100%); and in 2013, the low vole year, 121 nest boxes were occupied (34%), 98 of which successfully fledged young (81% of occupied boxes). Note that we could not successfully trap adults in all these nest boxes, which is why sample sizes differ from the data set used in this study.

We had a complete dataset on body condition, blood parasite infection and individual genetic heterozygosity from 448 adults (n_(2011)_ = 139, n_(2012)_ = 163 and n_(2013)_ = 151) with no repeated measurements (i.e., different breeding adults sampled throughout the study period), obtained from 190 different nest-boxes over 3 years. The number of first year breeders was low, especially during the low vole year (n_(2011)_ = 34, n_(2012)_ = 31 and n_(2013)_ = 6).

**Fig. 2:**
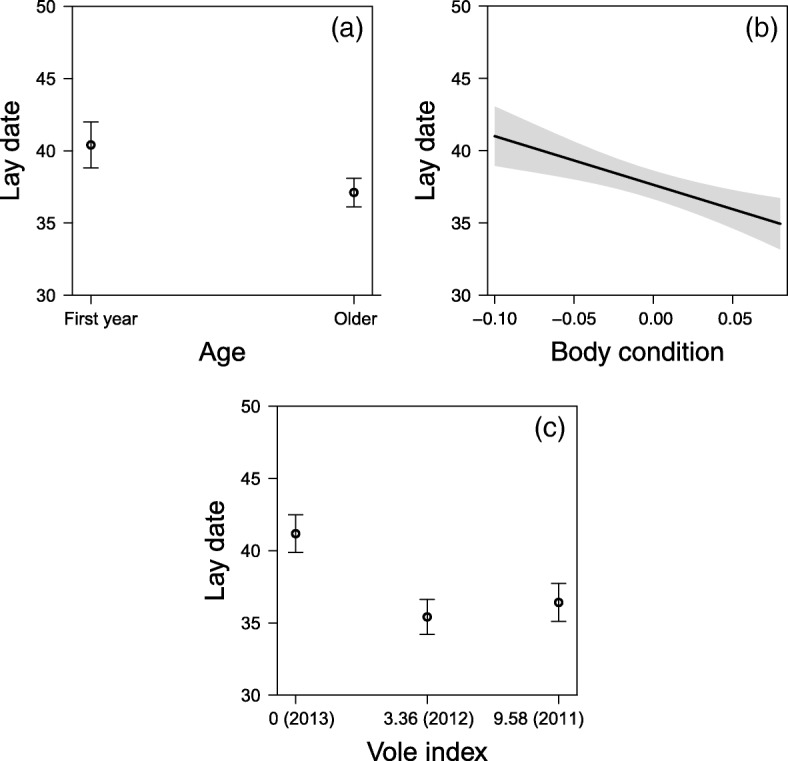
Variation in individual quality, vole abundance and territory land cover heterogeneity (Simpson’s Index) influencing the timing of breeding: (**a**) + 1-year parents (older adults); (**b**) individuals in higher body condition; and, (**c**) during years of higher vole abundance start egg-laying earlier (note the panel order ranges from the low vole year (2013) to the decrease (2012) and increase (2011) phase of the vole cycle). Plotted effect sizes plus 95% CIs; model details given in Table 1

The initiation of egg-laying (lay date) was determined by the age of the breeding adult, individual body condition and the phase of the vole cycle (Table 1). Egg-laying was on average 3.29 ± 0.78 SE days earlier in older parents compared to first-year breeders (Fig. 2a; least-square means post-hoc contrast: t-ratio = 4.22, *P* < 0.001). Individuals in higher body condition started egg-laying earlier than individuals in lower body condition (Fig. 2b). Egg-laying was initiated on average 5.76 ± 0.72 SE and 4.76 ± 0.78 SE days earlier during the increase and decrease phases (2011 and 2012), respectively, compared to the low phase (2013) of the vole cycle (Fig. 2c**)** (vole index = 0 and 3.36 post-hoc contrast: t-ratio = 8.05, *P* < 0.001; vole index = 0 and 9.58 post-hoc contrast: t-ratio = 6.11, *P* < 0.001; vole index = 3.36 and 9.58 post-hoc contrast: t-ratio − 1.41, *P* = 0.340).

**Table 1:** (a) Top models with ΔAIC_c_ < 4.0 for factors that influence the timing of breeding (Julian day of egg-laying) in Eurasian kestrels (all nests). (b) Model-averaged coefficients from a set of 2 models with ΔAIC_c_ < 4.0 (cumulative ω_i_ = 0.80) presented as estimated values ± (unconditional) SE, lower and upper 95% CIs, N containing models and relative variable importance (RVI); confidence intervals of parameter estimates not including zero in **bold**

(a)	Lay date (*n* = 438)	df	LogLik	AIC_c_	ΔAIC_c_	ω_i_
1.	age + voles + bc	7	− 536.72	1087.69	0.00	0.70
2.	age + voles + TLCH + bc	8	−537.60	1091.53	3.84	0.10
(b) Lay date (*n* = 438)	Estimate	SE	LCI	UCI	N	RVI
age	**−0.42**	**0.10**	**−0.62**	**− 0.23**	**2**	**1.00**
bc	**−0.13**	**0.04**	**−0.20**	**−0.06**	**2**	**1.00**
vole index (linear)	**−0.43**	**0.07**	**−0.57**	**−0.28**	**2**	**1.00**
vole index (quadratic)	**0.35**	**0.06**	**0.23**	**0.48**	**2**	**1.00**
TLCH	0.09	0.04	−0.04	0.21	1	0.13
*(Intercept)*	*0.42*	*0.10*	*0.28*	*0.74*	*–*	*–*

Age = 1^st^ year breeder or older, bc = body condition index, TLCH = territory land cover heterogeneity, voles = *Microtus* sp. vole index in spring (ordered factor)

**Table 2:** (a) Top models with ΔAIC_c_ < 4.0 for factors that influence nestling survival in Eurasian kestrels (successful nests only). (b) Model-averaged coefficients from a set of 7 models with ΔAIC_c_ < 4.0 (cumulative ω_i_ = 0.53) presented as estimated values ± (unconditional) SE, lower and upper 95% CIs, N containing models and relative variable importance (RVI); confidence intervals of parameter estimates not including zero in **bold**

(a)	Nestling survival (*n* = 428)	df	LogLik	AIC_c_	ΔAIC_c_	ω_i_
1.	ld cen + age + voles + TLCH + age × voles + age × TLCH + voles × TLCH	12	− 509.00	1042.75	0.00	0.18
2.	ld cen + age + voles + TLCH + age × voles + voles × TLCH	11	− 510.14	1042.92	0.17	0.17
3.	ld cen + voles + TLCH + voles × TLCH	8	− 514.57	1045.48	2.72	0.05
4.	ld cen + age + voles + TLCH + voles × TLCH	9	− 513.58	1045.59	2.83	0.04
5.	ld cen + age + voles + TLCH + dist + NND + age × voles + age × TLCH + voles × TLCH	14	− 508.60	1046.21	3.46	0.03
6.	ld cen + age + voles + TLCH + age × TLCH + voles × TLCH	10	− 512.88	1046.28	3.53	0.03
7.	ld cen + age + voles + TLCH + dist + NND + age × voles + voles × TLCH	13	− 509.71	1046.29	3.54	0.03
(b) Nestling survival (n = 428)	Estimate	SE	LCI	UCI	N	RVI
age	0.17	0.23	−0.29	0.64	6	0.91
vole index (linear)	0.21	0.41	−0.59	1.01	7	1.00
vole index (quadratic)	−0.02	0.42	−0.84	0.80	7	1.00
TLCH	0.23	0.17	−0.11	0.57	7	1.00
ld cen	**−0.35**	**0.08**	**−0.51**	**−0.18**	**7**	**1.00**
age × vole index (linear)	0.24	0.41	−0.65	1.14	4	0.77
age × vole index (quadratic)	**−0.89**	**0.48**	**−1.57**	**−0.21**	**4**	**0.77**
age × TLCH	0.27	0.18	−0.09	0.62	3	0.46
TLCH × vole index (linear)	0.17	0.13	−0.08	0.42	7	1.00
TLCH × vole index (quadratic)	**−0.33**	**0.13**	**−0.58**	**−0.07**	**7**	**1.00**
dist	0.08	0.04	−0.10	0.26	2	0.12
NND	−0.03	0.03	−0.20	0.14	2	0.12
*(Intercept)*	*1.65*	*0.23*	*1.19*	*2.10*	*–*	*–*

Age = 1^st^ year breeder or older, ld cen = lay date centred to the mean of the study year, vole index = *Microtus* sp. vole index in spring (ordered factor), TLCH = territory land cover, dist = distance to the closest forest edge (log transformed), NND = nearest neighbour distance (log transformed), “×” = indicating an interaction term

**Fig. 3:**
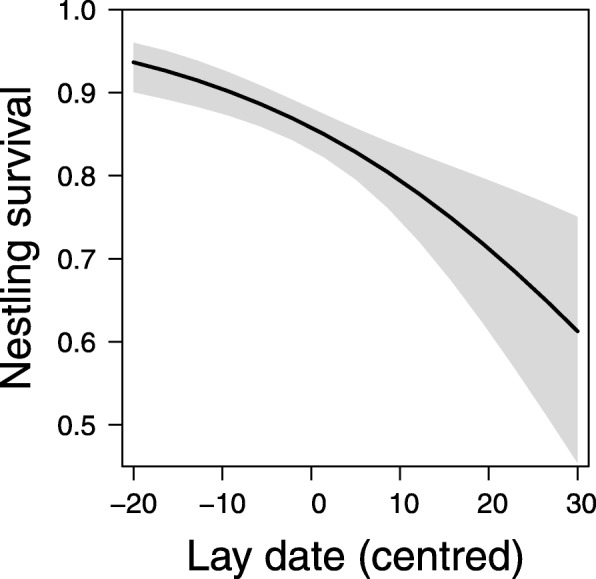
Variation in lay date (centred to the mean of the study year) influencing nestling survival. Plotted effect sizes plus 95% CIs; model details given in Table 2

**Fig. 4:**
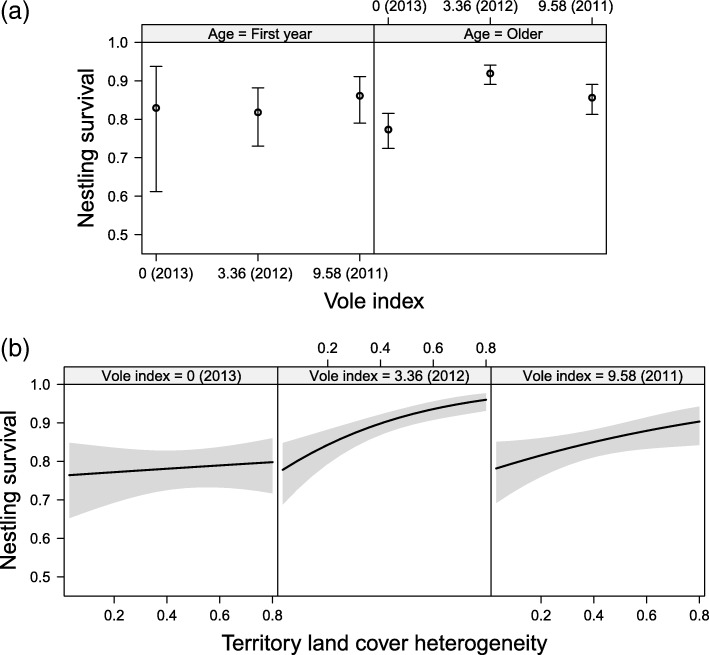
The interaction between (**a**) the age of the breeding adult and the vole cycle; and (**b**) territory land cover heterogeneity (Simpson’s Index) and the vole cycle influencing nestling survival. Plotted effect sizes plus 95% CIs; model details given in Table 2

We found higher nestling survival (Table 2) in earlier nests (Fig. 3) and during the decrease (2012) phase compared to the increase (2011) and low (2013) phases of the vole cycle, but this relationship was only evident in older breeders (vole index = 3.36, first year and older post-hoc contrast: odd ratio 0.396 ± 0.11 SE, z-ratio = − 3.21, *P* = 0.001; Fig. 4a, all least square mean post-hoc contrasts in Additional file 1: S9). The slope of the relationship between nestling survival and the vole cycle was further dependent on territory land cover heterogeneity, in a way that nestling survival was higher in more heterogeneous landscapes (small fields in the East) than in the more homogeneous landscapes (large fields in the West) during the increase (2011) and decrease (2012) phases of the vole cycle. However, this difference was not evident during the low vole year (2013, Fig. 4b).

## Discussion

We addressed variation in individual parental quality, vole abundance and land cover heterogeneity that induced differential onset of breeding (reflecting settlement decisions) and nestling survival (reflecting breeding investment versus breeding output; i.e., fitness consequences). The most interesting finding of our study was a strong positive effect of landscape heterogeneity on nestling survival of kestrels, but only when voles were relatively abundant, whereas a difference in the timing of breeding related to territory landscape heterogeneity was not evident.

### Sequential settlement

As expected, laying dates in our study population were positively correlated to vole abundance as egg-laying initiated earlier during the increase and decrease years of the vole cycle, as shown in previous studies [30, 50, 65]. Egg laying was also earlier in older breeders and those with higher body condition, following the sequential settlement paradigm [5]. Earlier egg-laying in older individuals (reflecting higher individual quality; see for instance [105]) have already been found in long-term data sets in our study population [50] and elsewhere [38, 106]. However, contrary to predictions i) and ii), landscape heterogeneity did not influence laying dates at the territory scale. Therefore, early individuals do not seem to settle preferentially in homogeneous landscapes where snow melts earlier. Nevertheless, landscape heterogeneity influenced nestling survival depending on local vole abundance, i.e. nestling survival was higher in more heterogeneous landscapes, but only during the increase and decrease phases of the vole cycle. These results are contradictory to the predictions iii) and iv), suggesting that heterogeneous habitats did not buffer against low abundance of the main prey.

We offer two non-mutually exclusive explanations for this pattern. It may be that i) higher alternative prey abundance boosts nestling survival in these heterogeneous landscapes when voles are abundant; or ii) that there are unexpected fine-scale spatial differences in vole abundance between homogenous and heterogeneous landscapes. Thus, when voles are abundant throughout the study area as shown in our long-term vole cycle data, they might reach even higher densities in heterogeneous territories, a phenomenon that has thus far been shown in western Europe [107].

More diverse prey communities in heterogeneous landscapes might enhance the reproductive performance of kestrels breeding in these territories when voles are abundant. Kestrels in our study area feed mainly on voles (46% of prey number between 1972 and 1983), followed by insects, shrews, birds, mice, lizards and frogs [43]. This author showed that diet composition depends on the vole cycle, but also on landscape composition, as more shrews and birds were caught in heterogeneous landscapes than in homogenous landscapes. For example, fledglings of forest passerines (mainly *Turdus* spp.) are an important alternative prey species particularly during low vole abundance years [43, 108].

### Maladaptive habitat decision-making

Our results highlighted that kestrels do not choose their territory according to its true value at the time of settlement. For example, egg-laying should be earlier in more heterogeneous landscapes, where nestling survival is highest during high vole abundance years, which was not the case. Although high-quality individuals settle earlier, they seem to settle randomly throughout the study area, at least over the 3-year study period. This suggests that large-scale spatial synchrony in vole fluctuations [69] is the main factor constraining settlement decisions in nomadic avian predators (see refs on dispersal of nomadic raptors, [71, 80, 109]). However, there might be, as stated above, fine-scale differences in vole abundance that could drive the higher nestling survival in heterogeneous landscapes when vole abundance is generally high to medium (2011 and 2012). The fact that there was no difference in nestling survival between heterogeneous landscapes and homogenous landscapes when vole abundance was generally low (2013), shows that these habitats do not sufficiently buffer against low main prey densities.

Maladaptive habitat decision-making implies a preference for low-quality habitats over high-quality options causing the species to fall into what is known as an ecological trap (reviewed in [11, 110–113]). Our results appeared not to support the ecological trap hypothesis as no spatial variations in laying dates (as a proxy of settlement decision) depending on landscape heterogeneity could be detected. However, our results strikingly indicate that landscape homogenization linked to agricultural intensification disrupts the expected positive effect of vole abundance on reproductive success of kestrels (see Fig. 4b; nestling survival had similar low predicted values in very homogeneous landscapes in the three different years of the vole cycle).

### Effects of different individual quality indices on reproductive performance

The measurement we were specifically interested in was nestling survival, i.e. the clutch size per fledgling ratio, because this variable reflects individual variations in reproductive performance linked to parental quality. We found clear evidence that older breeders performed better in raising their offspring than first year inexperienced breeders, but this was only true during the increase phase of the vole cycle. This might indicate that learning about fluctuating food conditions, and taking advantage of the periodically high vole abundance, is part of the breeding experience that older individuals already gained, similarly to the development of migratory behaviour in the Black Kite *Milvus migrans* [114]. Additionally, the number of first year breeders in kestrels is especially low during the low vole year [50], which also underlines the importance of breeding experience to persist under such fluctuating food conditions.

Finally, our fifth prediction, i.e. that short-term individual quality measurements and breeding experience (parental age) have a stronger influence under such fluctuating food conditions than long-term individual quality measurements, was fully met. We indeed found body condition, a highly seasonal estimate, to modify the timing of breeding, but blood parasite infection or individual genetic heterozygosity did not feature into any of the top models. This is also partly in line with our prediction (iv), that those individuals that are in good body condition (high body mass relative to body size) after migration will be able to secure a territory and successfully raise fledglings, but this was not linked to landscape habitat heterogeneity as we expected.

## Conclusions

Early arriving kestrels that are older and/or in higher body condition started egg-laying earlier, but did not show a clear habitat preference for either homogeneous agricultural landscapes or more heterogeneous landscapes. However, kestrels breeding in the latter had in fact higher nestling survival than their con-specifics, an effect that was visible only during the increase and decrease phases of the vole cycle. Therefore, the benefit of breeding in heterogeneous habitats that offer alternative prey is not enough to compensate for low vole abundances, as there was no difference in nestling survival between sites in the year of vole scarcity (2013). Unexpectedly, differences in nestling survival between habitats and depending on vole abundance were not mirrored by spatial variations in egg-laying dates. This suggests that other factors are of essence in this kestrel population. For example, large-scale breeding dispersal aiming to track cyclic fluctuations in vole abundance might be more important in determining settlement timing and decisions than landscape heterogeneity. However, landscape heterogeneity appears as a main driver of high reproductive performance under favourable food conditions.

Overall, for rodent specialist predators, the high reproductive performance achieved during years of high food abundance is essential to achieve high lifetime reproductive performance [115] and to maintain the whole population dynamics at large-scale [116]. Our results indicate that maintaining these heterogeneous agricultural habitats (probably correlated with lower agricultural intensification), allowing kestrels to take full advantage of vole peak abundance are essential for the conservation of this farmland raptor and potentially many other species dependent of agro-ecosystems (e.g.; [117]). Our findings have important implications for biodiversity conservation in agricultural landscapes, since the loss of ecological heterogeneity at multiple spatial and temporal scales is a universal consequence of agricultural intensification and a key threat to biodiversity in farmland areas [118, 119].

## Additional file

Additional file 1: **Table S1.** Table of habitats found in the study area and Eurasian kestrel territories. **Figure S2.** Figure showing the relationship between territory land cover heterogeneity and percentage of farmland area in kestrel territories. **Figure S3.** Figure showing the periodic 3-year vole cycle over the entire long-term survey period from 1973 to 2015. **S4 Material and Methods.** Blood parasite infection. **Table S5.** Table of Primer set Falconidae: Repeat motif and primer sequences for 24 microsatellites. **S6 Material and Methods.** Individual genetic heterozygosity. **Table S7.** Candidate list for parameters influencing the timing of breeding (Julian day of egg-laying) and nestling survival (ratio of eggs that fledged successfully) in Eurasian kestrels; and complete model list following AICc ranking and model weights. **Figure S8.** Figure of spatial distribution of ‘individual quality’ indices quantified in male and female kestrels. **Figure S9.** Figure of least square means post-doc contrasts of the interaction term age of the breeding adult, and the vole cycle, influencing nestling survival in Eurasian kestrels. (PDF 508 kb)

Additional file 2: Supporting data. (XLSX 82 kb)

## Data Availability

Morphological and ringing data on Eurasian Kestrels have been provided to the Finnish Ringing Scheme (https://www.luomus.fi/en/bird-ringing). All supporting data are available as supplementary material enclosed to this publication (Additional file 2).
