# Supplementary material for: Landscape homogenization due to agricultural intensification disrupts the relationship between reproductive success and main prey abundance in an avian predator
Source: Front Zool. 2019 Aug 6;16:31. doi: 10.1186/s12983-019-0331-z (PMC6683578; doi:10.1186/s12983-019-0331-z)
Supplement: Supplementary file 1 — Table S1. Table of habitats found in the study area and Eurasian kestrel territories. Figure S2. Figure showing the relationship between territory land cover heterogeneity and percentage of farmland area in kestrel territories. Figure S3. Figure showing the periodic 3-year vole cycle over the entire long-term survey period from 1973 to 2015. S4 Material and Methods. Blood parasite infection. Table S5. Table of Primer set Falconidae: Repeat motif and primer sequences for 24 microsatellites. S6 Material and Methods. Individual genetic heterozygosity. Table S7. Candidate list for parameters influencing the timing of breeding (Julian day of egg-laying) and nestling survival (ratio of eggs that fledged successfully) in Eurasian kestrels; and complete model list following AICc ranking and model weights. Figure S8. Figure of spatial distribution of ‘individual quality’ indices quantified in male and female kestrels. Figure S9. Figure of least square means post-doc contrasts of the interaction term age of the breeding adult, and the vole cycle, influencing nestling survival in Eurasian kestrels. (PDF 508 kb) [file 12983_2019_331_MOESM1_ESM.pdf]

Additional file 1

Landscape homogenization due to agricultural intensification disrupts the relationship between reproductive success and main prey abundance in an avian predator

Petra Sumasgutner, Julien Terraube, Aurélie Coulon, Alexandre Villers, Nayden Chakarov, Luise Kruckenhauser and Erkki Korpimäki

Doi: 10.1186/s12983-019-0331-z

S1 Table: Habitats found in the study area and Eurasian kestrel territories; percentages in the whole study area, their average around kestrel territories, and separated for the three study years.

**landuse***	**study area**	**kestrel territories**	**kestrel 2011** (buffer r=650m)	**kestrel 2012** (buffer r=694m)	**kestrel 2013** (buffer r=843m)
birch dominated clear cuts	18.80	7.93	6.45	6.80	9.49
young birch dominated	1.20	0.74	0.71	0.62	0.82
young pine dominated	13.68	5.33	4.35	4.39	6.62
young spruce dominated	0.05	0.02	0.01	0.02	0.02
young birch pine	2.13	1.01	0.81	0.82	1.27
young spruce pine	0.47	0.18	0.16	0.17	0.23
young birch spruce	0.19	0.07	0.05	0.05	0.08
mature birch dominated	0.44	0.26	0.28	0.22	0.36
mature pine dominated	14.67	5.68	4.47	5.15	7.14
mature spruce dominated	0.09	0.03	0.03	0.03	0.03
mature birch pine	1.53	0.71	0.65	0.60	0.83
mature pine spruce	1.29	0.44	0.35	0.41	0.58
mature birch spruce	0.22	0.08	0.06	0.06	0.08
old birch dominated	0.09	0.05	0.06	0.04	0.06
old pine dominated	3.42	1.65	1.58	1.55	1.90
old spruce dominated	0.73	0.33	0.24	0.34	0.37
old birch pine	0.42	0.19	0.19	0.20	0.19
old birch spruce	1.98	0.84	0.61	0.83	1.08
old birch spruce	0.06	0.02	0.01	0.02	0.02
build up	1.77	0.97	0.76	0.85	1.22
roads	2.16	1.88	1.77	1.85	2.09
mines	0.09	0.12	0.25	0.13	0.06
peatland bogs and similar	5.06	2.16	2.01	2.16	2.22
farmlands	28.30	68.56	73.56	71.97	62.60
waters	1.17	0.74	0.60	0.72	0.65

* in %

S2 Figure: Relationship between territory land cover heterogeneity (TLCH, ranging 0-1) and percentage of farmland area (20-100%) in kestrel territories. Homogeneous open habitat in the West of the study area indicates agricultural fields (low TLCH values) and heterogeneous habitat in the East indicates a mosaic landscape of different habitat, including up to 50% agricultural fields.

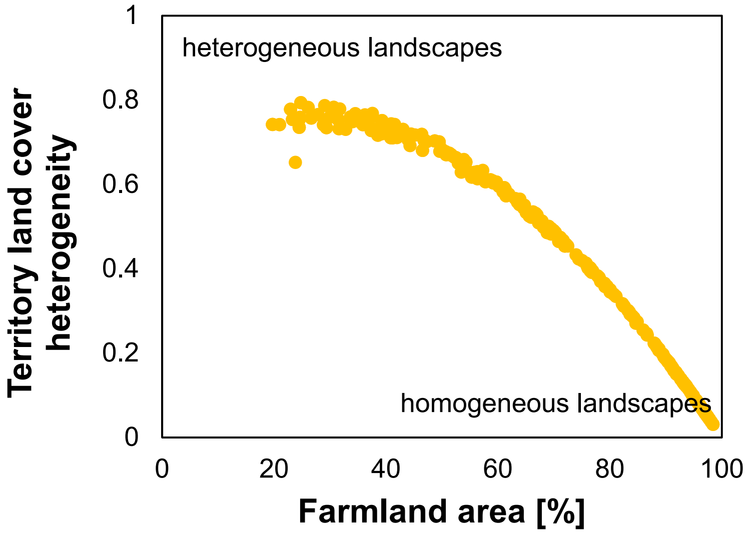

S3 Figure: Periodic 3-year vole cycle over the entire long-term survey period from 1973 to 2015 based on snap-trapping data (no. of Microtus voles trapped per 100 trap-night) in spring (May) and autumn (Sep), in four sampling plots located in the study area in the Kauhava region, Western Finland (data from Korpimäki and Hakkarainen 2012; and Korpimäki E. unpublished data).

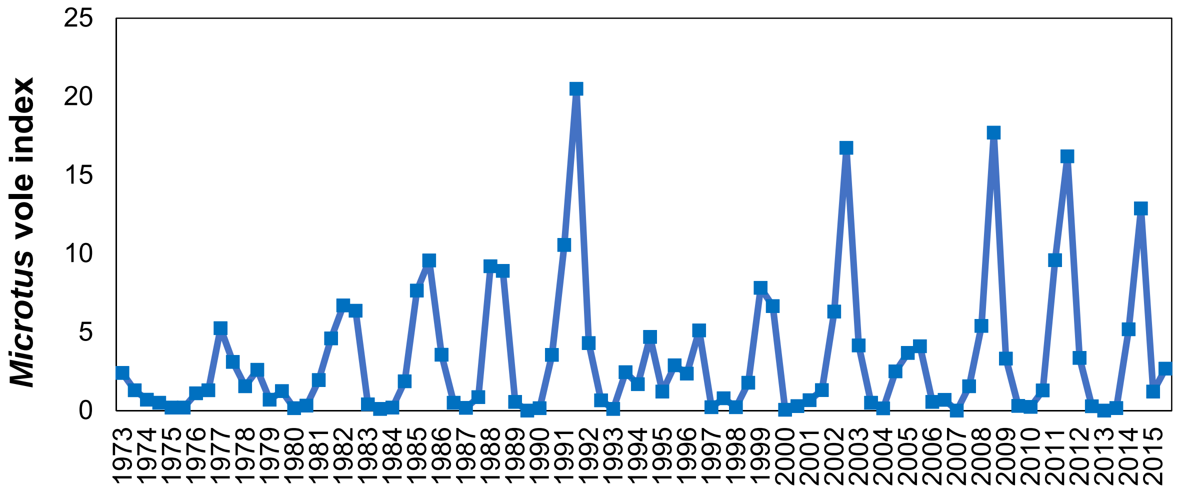

S4 Material and Methods: *Blood parasite infection*

DNA extractions were done in the Center of Evolutionary Applications, University of Turku, Finland, using the salt extraction method modified from Aljanabi and Martinez (1997). DNA concentrations in ng/µl were measured using Nanodrop. Kestrel DNA samples were scored for blood parasites of the genera *Haemoproteus*, *Plasmodium* and *Leucocytozoon* at the Molecular Ecology and Evolution Lab, Lund University, Sweden. We used a nested PCR with the primer pairs HAEMF/HAEMR2 (H&P) or HAEMFL/HAEMR2L (L), following a first amplification round with the primers HaemNFI/HAEMNR3 (Hellgren, Waldenström & Bensch 2004; Bensch, Hellgren & Pérez-Tris 2009). No samples were found positive for *Leucocytozoon*. Out of the samples positive for *Haemproteus/Plasmodium*, amplicons of the mitochondrial cytochrome B gene were Sanger sequenced for 46 infections. Of these, 45 cases were infected with the specific *Haemoproteus nisi* lineage H-FATI1 (LK3) and one case was infected with the widely generalistic *Plasmodium circumflexum* lineage SW5 (Bensch, Hellgren & Pérez-Tris 2009; Valkiūnas *et al.* 2014). We only used *Haemoproteus* infection risk (presence/absence in an individual adult) as variable for blood parasite infection in the analyses.

S5 Table: Primer set *Falconidae*: Repeat motif and primer sequences for 24 microsatellites, established for *F. peregrinus* and *F. naumanni*. Size range, number of alleles, observed heterozygoty (HO) and expected heterozygoty (HE) based on cross-amplification in 448 adult Eurasian kestrels sampled between 2011 and 2013 in the Kauhava-Lapua region, Finland.

Locus	GenBank Accession no.	Repeat motif (bases)	Primer sequences 5'–3'	Range in size (bp)	Number of alleles	H_O	H_E	Literature	Multiplex nb & dye
NVH fp5	AF118420	$(GT)_{11}$	F:CCGTTCTGGAGTCAAAAC<br>R:CATGCAGCACTTTATTCAG	94-124	15	0.78	0.86	(Nesje et al. 2000)	2 - VIC
NVH fp31	AF118422	$(CA)_{17}$	F:ATCACCTGCACATAGCTG<br>R:TTTAGCTCCTCTCTCTCAC	132-148	9	0.45	0.62	(Nesje et al. 2000)	1 - ATTO550
NVH fp46-1	AF118423	$(CA)_{11}$	F:TTAGCCTCGCAGCTTCAG<br>R:GTAATGAAAAGTCTTTGGGG	112-136	11	0.65	0.77	(Nesje et al. 2000)	1 - Yakima Yellow
NVH fp82-2	AF118428	$(GT)_{10}$	F:CTGCACGAGGAGATGATG<br>R:CCAGATAGCTGTGAAATGG	124-144	10	0.69	0.78	(Nesje et al. 2000)	1 - ATTO565
NVH fp86-2	AF118429	$(CA)_{11}$	F:GTAAATAAGCCTCCAAAAGG<br>R:CATGCTTCCTGATTACTTC	140-156	9	0.67	0.73	(Nesje et al. 2000)	5 - ATTO550
NVH fp89	AF118430	$(AT)_{12}$	F:CTCTGCCCTGAATACTTAC<br>R:GAATCTTGTTTGCATTGGAG	112-142	9	0.63	0.63	(Nesje et al. 2000)	4 - Yakima Yellow
NVH fp107	AF118434	$(GT)_{11}TT(GT)_8$	F:ACAGATTTGATTGCCAGG<br>R:TGCCATGTCACATTCATAC	175-249	29	0.64	0.93	(Nesje et al. 2000)	5 - Yakima Yellow
Fnd1.1	FM180205	$(TC)_{10}$	F: TCACCCTGCTTCTTGCTTCT<br>R: CCAAACCTCTCTTTCCCAGA	235-263	11	0.43	0.48	(Padilla et al. 2009)	4 - PET
Fnd1.3	FM180207	$(AG)_5$	F: GCCTAAGGTTTCCCTCAGCTA<br>R: TCATCAGACTGCAAAACTGGA	175-233	21	0.80	0.87	(Padilla et al. 2009)	2 - 6FAM
Fnd1.4	FM180208	$(AG)_{11}$	F: AGCTGAAGCCAACCTGGTAA<br>R: CATGTCCTTGCCTGAGGAAC	220-238	7	0.35	0.37	(Padilla et al. 2009)	4 - 6FAM
Fnd1.5	FM180210	$(TC)_{15}$	F: CCATTGATTTTCATCAACTACA<br>R: CCTGTTGAGAATGCGTGAAA	211-245	17	0.78	0.78	(Padilla et al. 2009)	3 - VIC

Locus	GenBank Accession no.	Repeat motif (bases)	Primer sequences 5'–3'	Range in size (bp)	Number of alleles	H_O	H_E	Literature	Multiplex nb & dye
Fnd1.6	FM180211	$(GATG)_{13}$ N_4 $(GATG)_3$ N_{30} $(GA)_{13}$	F: ATTTGTGGCAAACCAGAGGA<br>R: CCCACATTTTCCAAACAAGG	289-363	36	0.92	0.93	(Padilla et al. 2009)	3 - NED
Fnd1.7	FM180212	$(CT)_{10}$	F: TACCGTCCTTGTTCGGAAGT<br>R: CTACAGTCTGCCCCCAAGAA	223-245	10	0.68	0.68	(Padilla et al. 2009)	5 - 6FAM
Fnd1.8	FM180213	$(TC)_{12}$	F: CAGTGACGCCTGAAAGATGA<br>R: GCTTGGAAAGTCCTCTGCTG	180-200	9	0.37	0.72	(Padilla et al. 2009)	5 - PET
Fnd2.1	FM180214	$(TG)_9$	F: AGTCATGGCTTCCGATCAAG<br>R: TCAGGCAGCCTTATTTTTGG	185-213	15	0.88	0.88	(Padilla et al. 2009)	4 - VIC
Fnd2.2	FM180215	$(CA)_8$ $(TA)_3$	F: AACTTTGCCCCAGATCACAC<br>R: GCACAGAGACCCCGTTACAT	174-200	14	0.55	0.54	(Padilla et al. 2009)	2 - NED
Fnd2.3	FM180216	$(AC)_{12}$	F: CAAGCAGGGTGAAAATCCAT<br>R: GTTTTCCCTCATTGCCTGAA	211-251	14	0.69	0.67	(Padilla et al. 2009)	5 - NED
Fnd2.4	FM180217	$(AT)_{10}$ N_{23} $(CA)_5$ AG $(CA)_4$	F: ACCACAGGTGCTTTTTCACA<br>R: AAAAGAAATGGTGGCAGGTG	160-186	14	0.47	0.93	(Padilla et al. 2009)	5 - ATTO550
Fnd2.5	FM180218	$(CA)_{12}$ $(TA)_4$	F: CACTACCAGCCCTGAACCAT<br>R: CTTCTTGACAGGGGTGTGGT	207-235	14	0.94	0977	(Padilla et al. 2009)	3 - PET
Fnd2.6	FM180219	$(GT)_8$	F: TCCGGTGTTACATTCCCATT<br>R: AAGCCCCTTTCTACACAGCA	202-238	20	0.61	0.74	(Padilla et al. 2009)	4 - 6FAM
Fn1-11	EF152566	$(AG)_{15}$	F:TTCTATTGTAGGAATCCTGGAAACTT<br>R:GGCTGTTATTTATTGGAAGAGTGA	177-673	85	0.94	0.97	(Ortego et al. 2007)	1 - 6FAM
Fn2-14	EF152565	$((TCTA)_X A)_Y$	F:TTGCCAGCTTTTGAACCCTAA<br>R:AAATTCAGGCCACCCACATC	134-242	26	0.47	0.93	(Ortego et al. 2007)	1 - ATTO550

S6 Material and Methods*: Individual genetic heterozygosity*

All following laboratory work was done in the Central Laboratories of the Natural History Museum Vienna, Austria. We used 22 different microsatellites established for *F. peregrinus* (Nesje *et al.* 2000) and *F. naumanni* (Ortego *et al.* 2007; Padilla *et al.* 2009): *NVH fp5, NVH fp31, NVH fp46-1, NVH fp82-2, NVH fp86-2, NVH fp89, NVH fp107, Fnd1.1, Fnd1.3, Fnd1.4, Fnd1.5, Fnd1.6, Fnd1.7, Fnd1.8, Fnd2.1, Fnd2.2, Fnd2.3, Fnd2.4, Fnd2.5, Fnd2.6, Fn1-11, Fn2-14.* PCR reactions were performed using the QIAGEN Multiplex PCR Kit with 20-60 ng/µl DNA following the standard protocol. PCR cycles consisted of an initial activation step at 95°C for 15 min (HotStartTaq DNA Polymerase) and 35 cycles of 94°C for 30 sec, 48°C-57°C annealing temperature for 90 s and 72°C for 90 s, followed by a final extension of 72°C for 20 min. Differences in final allele sizes and in fluorescent dye labels of primers allowed for pooling multiple loci in 5 different marker sets for multiplex PCR. The pooled products were then diluted with water, mixed with HiDiFormamid (Applied Biosystems) and internal size standard LIZ500 and run on an ABI3130x1sequencer. All loci were visually identified using the program ABI PeakScanner 1.0 by the same person (Author A). Final allele sizes were determined using the binning software Tandem 1.01 (Matschiner & Salzburger 2009). We used the software GENETIX 4.05 (Belkhir *et al.* 2004) to identify outliers and MICRO-CHECKER 2.2.3 (Van Oosterhout *et al.* 2004) to test for potential scoring errors, allelic dropout and null alleles; the markers *Fn2-14*, *fn1.8* and *fp107* were excluded thereafter due to null alleles. We only included samples where >70% of the markers successfully amplified. Ambiguous samples were re-genotyped up to three times. CERVUS 3.0 (Kalinowski, Taper & Marshall 2007), GENEPOP 4.2 (Raymond & Rousset 1995; Rousset 2008) and the R package 'PopGenReport' (Adamack & Gruber 2014) were used to determine i) departures from Hardy-Weinberg equilibrium at each locus, with adjusted P-values for multiple comparisons using false discovery rate (FDR) to control for multiple testing (Achard 2012); and ii) genotypic linkage disequilibrium for each pair of loci; the markers *fp82-2* (significantly linked to *fp46-1*) and *fp86-2* (significantly linked to *fn2.4*) were excluded thereafter. The remaining 17 microsatellites were used to estimate five commonly used measures of individual multilocus heterozygosity using the GENHET R function (Author C 2010). We chose the standardized heterozygosity (Chapman *et al.* 2009), as the variable 'Hs_exp', to be used as an index for individual genetic heterozygosity in the analyses thereafter.

S7 Table: a) Candidate list for parameters influencing the timing of breeding (Julian day of egg-laying) and nestling survival (ratio of eggs that fledged successfully) in Eurasian kestrels *Falco tinnunculus:* breeding experience (age), individual quality [either body condition (bc), *Haemoproteus* sp. infection risk (haem), individual genetic heterozygosity (Hs_exp)], territory land cover heterogeneity (Simpson's index, TLCH) and vole abundance in spring (voles). Additional co-variates were the distance to the closest forest edge (dist) and nearest neighbour distance (NND), both log-transformed. The only difference to the candidate list for nestling survival is that we additionally fitted lay date (centred to the mean of the study year) because of a known strong correlation between egg-laying and the vole cycle. Information theoretic approach followed by model averaging of candidate models with ΔAICc < 4. Nest box ID was used as random term.

m1 (null model)									
m2 (yearly variation only – i.e., vole index only					voles +				
m3						TLCH +			
m4								dist	
m5									NND
m6					voles +	TLCH +			
m7					voles +			dist	
m8					voles +				NND
m9					voles +	TLCH +		dist +	NND
m10					voles +	TLCH +	voles : TLCH +		
m11					voles +	TLCH +	voles : TLCH +	dist +	NND
m12	age +				voles +				
m13	age +				voles +	TLCH +			
m14	age +				voles +	TLCH +		dist +	NND
m15	age +				voles +	TLCH +	voles : TLCH +		
m16	age +				voles +	TLCH +	voles : TLCH +	dist +	NND
m17	age +		age : voles +		voles +				
m18	age +		age : voles +		voles +	TLCH +			
m19	age +		age : voles +		voles +	TLCH +		dist +	NND
m20	age +		age : voles +		voles +	TLCH +	voles : TLCH +		
m21	age +		age : voles +		voles +	TLCH +	voles : TLCH +	dist +	NND
m22	age +			age : TLCH +	voles +	TLCH +			
m23	age +			age : TLCH +	voles +	TLCH +		dist +	NND
m24	age +			age : TLCH +	voles +	TLCH +	voles : TLCH +		
m25	age +			age : TLCH +	voles +	TLCH +	voles : TLCH +	dist +	NND
m26	age +		age : voles +	age : TLCH +	voles +	TLCH +			
m27	age +		age : voles +	age : TLCH +	voles +	TLCH +		dist +	NND
m28	age +		age : voles +	age : TLCH +	voles +	TLCH +	voles : TLCH +		
m29	age +		age : voles +	age : TLCH +	voles +	TLCH +	voles : TLCH +	dist +	NND
m30	age +	Hs_exp +			voles +				
m31	age +	Hs_exp +			voles +	TLCH +			

m32	age +	Hs_exp +			voles +	TLCH +		dist +	NND
m33	age +	Hs_exp +			voles +	TLCH +	voles : TLCH +		
m34	age +	Hs_exp +			voles +	TLCH +	voles : TLCH +	dist +	NND
m35	age +	Hs_exp +	Hs_exp : voles +		voles +				
m36	age +	Hs_exp +	Hs_exp : voles +		voles +	TLCH +			
m37	age +	Hs_exp +	Hs_exp : voles +		voles +	TLCH +		dist +	NND
m38	age +	Hs_exp +	Hs_exp : voles +		voles +	TLCH +	voles : TLCH +		
m39	age +	Hs_exp +	Hs_exp : voles +		voles +	TLCH +	voles : TLCH +	dist +	NND
m40	age +	Hs_exp +		Hs_exp : TLCH +	voles +	TLCH +			
m41	age +	Hs_exp +		Hs_exp : TLCH +	voles +	TLCH +		dist +	NND
m42	age +	Hs_exp +		Hs_exp : TLCH +	voles +	TLCH +	voles : TLCH +		
m43	age +	Hs_exp +		Hs_exp : TLCH +	voles +	TLCH +	voles : TLCH +	dist +	NND
m44	age +		Hs_exp : voles +	Hs_exp : TLCH +	voles +	TLCH +			
m45	age +		Hs_exp : voles +	Hs_exp : TLCH +	voles +	TLCH +		dist +	NND
m46	age +		Hs_exp : voles +	Hs_exp : TLCH +	voles +	TLCH +	voles : TLCH +		
m47	age +		Hs_exp : voles +	Hs_exp : TLCH +	voles +	TLCH +	voles : TLCH +	dist +	NND
m48		Hs_exp +			voles +				
m49		Hs_exp +			voles +	TLCH +			
m50		Hs_exp +			voles +	TLCH +		dist +	NND
m51		Hs_exp +			voles +	TLCH +	voles : TLCH +		
m52		Hs_exp +			voles +	TLCH +	voles : TLCH +	dist +	NND
m53		Hs_exp +	Hs_exp : voles +		voles +				
m54		Hs_exp +	Hs_exp : voles +		voles +	TLCH +			
m55		Hs_exp +	Hs_exp : voles +		voles +	TLCH +		dist +	NND
m56		Hs_exp +	Hs_exp : voles +		voles +	TLCH +	voles : TLCH +		
m57		Hs_exp +	Hs_exp : voles +		voles +	TLCH +	voles : TLCH +	dist +	NND
m58		Hs_exp +		Hs_exp : TLCH +	voles +	TLCH +			
m59		Hs_exp +		Hs_exp : TLCH +	voles +	TLCH +		dist +	NND
m60		Hs_exp +		Hs_exp : TLCH +	voles +	TLCH +	voles : TLCH +		
m61		Hs_exp +		Hs_exp : TLCH +	voles +	TLCH +	voles : TLCH +	dist +	NND
m62			Hs_exp : voles +	Hs_exp : TLCH +	voles +	TLCH +			
m63			Hs_exp : voles +	Hs_exp : TLCH +	voles +	TLCH +		dist +	NND
m64			Hs_exp : voles +	Hs_exp : TLCH +	voles +	TLCH +	voles : TLCH +		
m65			Hs_exp : voles +	Hs_exp : TLCH +	voles +	TLCH +	voles : TLCH +	dist +	NND
m66	age +	haem +			voles +				
m67	age +	haem +			voles +	TLCH +			
m68	age +	haem +			voles +	TLCH +		dist +	NND
m69	age +	haem +			voles +	TLCH +	voles : TLCH +		

m70	age +	haem +			voles +	TLCH +	voles : TLCH +	dist +	NND
m71	age +	haem +	haem : voles +		voles +				
m72	age +	haem +	haem : voles +		voles +	TLCH +			
m73	age +	haem +	haem : voles +		voles +	TLCH +		dist +	NND
m74	age +	haem +	haem : voles +		voles +	TLCH +	voles : TLCH +		
m75	age +	haem +	haem : voles +		voles +	TLCH +	voles : TLCH +	dist +	NND
m76	age +	haem +		haem : TLCH +	voles +	TLCH +			
m77	age +	haem +		haem : TLCH +	voles +	TLCH +		dist +	NND
m78	age +	haem +		haem : TLCH +	voles +	TLCH +	voles : TLCH +		
m79	age +	haem +		haem : TLCH +	voles +	TLCH +	voles : TLCH +	dist +	NND
m80	age +		haem : voles +	haem : TLCH +	voles +	TLCH +			
m81	age +		haem : voles +	haem : TLCH +	voles +	TLCH +		dist +	NND
m82	age +		haem : voles +	haem : TLCH +	voles +	TLCH +	voles : TLCH +		
m83	age +		haem : voles +	haem : TLCH +	voles +	TLCH +	voles : TLCH +	dist +	NND
m84		haem +			voles +				
m85		haem +			voles +	TLCH +			
m86		haem +			voles +	TLCH +		dist +	NND
m87		haem +			voles +	TLCH +	voles : TLCH +		
m88		haem +			voles +	TLCH +	voles : TLCH +	dist +	NND
m89		haem +	haem : voles +		voles +				
m90		haem +	haem : voles +		voles +	TLCH +			
m91		haem +	haem : voles +		voles +	TLCH +		dist +	NND
m92		haem +	haem : voles +		voles +	TLCH +	voles : TLCH +		
m93		haem +	haem : voles +		voles +	TLCH +	voles : TLCH +	dist +	NND
m94		haem +		haem : TLCH +	voles +	TLCH +			
m95		haem +		haem : TLCH +	voles +	TLCH +		dist +	NND
m96		haem +		haem : TLCH +	voles +	TLCH +	voles : TLCH +		
m97		haem +		haem : TLCH +	voles +	TLCH +	voles : TLCH +	dist +	NND
m98			haem : voles +	haem : TLCH +	voles +	TLCH +			
m99			haem : voles +	haem : TLCH +	voles +	TLCH +		dist +	NND
m100			haem : voles +	haem : TLCH +	voles +	TLCH +	voles : TLCH +		
m101			haem : voles +	haem : TLCH +	voles +	TLCH +	voles : TLCH +	dist +	NND
m102	age +	bc +			voles +				
m103	age +	bc +			voles +	TLCH +			
m104	age +	bc +			voles +	TLCH +		dist +	NND
m105	age +	bc +			voles +	TLCH +	voles : TLCH +		
m106	age +	bc +			voles +	TLCH +	voles : TLCH +	dist +	NND
m107	age +	bc +	bc : voles +		voles +				

m108	age +	bc +	bc : voles +		voles +	TLCH +			
m109	age +	bc +	bc : voles +		voles +	TLCH +		dist +	NND
m110	age +	bc +	bc : voles +		voles +	TLCH +	voles : TLCH +		
m111	age +	bc +	bc : voles +		voles +	TLCH +	voles : TLCH +	dist +	NND
m112	age +	bc +		bc : TLCH +	voles +	TLCH +			
m113	age +	bc +		bc : TLCH +	voles +	TLCH +		dist +	NND
m114	age +	bc +		bc : TLCH +	voles +	TLCH +	voles : TLCH +		
m115	age +	bc +		bc : TLCH +	voles +	TLCH +	voles : TLCH +	dist +	NND
m116	age +		bc : voles +	bc : TLCH +	voles +	TLCH +			
m117	age +		bc : voles +	bc : TLCH +	voles +	TLCH +		dist +	NND
m118	age +		bc : voles +	bc : TLCH +	voles +	TLCH +	voles : TLCH +		
m119	age +		bc : voles +	bc : TLCH +	voles +	TLCH +	voles : TLCH +	dist +	NND
m120		bc +			voles +				
m121		bc +			voles +	TLCH +			
m122		bc +			voles +	TLCH +		dist +	NND
m123		bc +			voles +	TLCH +	voles : TLCH +		
m124		bc +			voles +	TLCH +	voles : TLCH +	dist +	NND
m125		bc +	bc : voles +		voles +				
m126		bc +	bc : voles +		voles +	TLCH +			
m127		bc +	bc : voles +		voles +	TLCH +		dist +	NND
m128		bc +	bc : voles +		voles +	TLCH +	voles : TLCH +		
m129		bc +	bc : voles +		voles +	TLCH +	voles : TLCH +	dist +	NND
m130		bc +		bc : TLCH +	voles +	TLCH +			
m131		bc +		bc : TLCH +	voles +	TLCH +		dist +	NND
m132		bc +		bc : TLCH +	voles +	TLCH +	voles : TLCH +		
m133		bc +		bc : TLCH +	voles +	TLCH +	voles : TLCH +	dist +	NND
m134			bc : voles +	bc : TLCH +	voles +	TLCH +			
m135			bc : voles +	bc : TLCH +	voles +	TLCH +		dist +	NND
m136			bc : voles +	bc : TLCH +	voles +	TLCH +	voles : TLCH +		
m137			bc : voles +	bc : TLCH +	voles +	TLCH +	voles : TLCH +	dist +	NND

b) Complete model list following AICc ranking and model weights (ωi) for the timing of breeding (Julian day of egg-laying) in Eurasian kestrels *Falco tinnunculus*. Breeding experience (age), individual quality [either body condition (bc), *Haemoproteus* sp. infection risk (haem), individual genetic heterozygosity (Hs_exp)], territory land cover heterogeneity (Simpson's index, TLCH) and vole abundance in spring (voles) were fitted as fixed effects. Additional co-variates were the distance to the closest forest edge (dist) and nearest neighbour distance (NND), both log-transformed. Nest box ID was used as random term. Model used for model averaging (ΔAICc < 4) in bold.

Model nb	Candidate models response variable: lay date	df	LogLik	AICc	ΔAICc	ωi
**1**	**age + voles + bc**	**7**	**-536.72**	**1087.69**	**0.00**	**0.70**
**2**	**age + voles + TLCH + bc**	**8**	**-537.60**	**1091.53**	**3.84**	**0.10**
3	age + voles + TLCH + bc + voles x TLCH	10	-536.06	1092.63	4.94	0.06
4	age + voles + bc + voles x bc	9	-537.18	1092.79	5.09	0.05
5	age + voles	6	-540.78	1093.75	6.06	0.03
6	age + voles + TLCH + bc + voles x bc	10	-538.13	1096.78	9.09	0.01
7	age + voles + TLCH	7	-541.67	1097.60	9.90	0.00
8	age + voles + TLCH + dist + NND + bc	10	-538.58	1097.67	9.97	0.00
9	age + haem + voles	7	-541.76	1097.78	10.09	0.00
10	age + voles + TLCH + bc + voles x TLCH + voles x bc	12	-536.59	1097.90	10.21	0.00
11	age + voles + age x voles	8	-541.00	1098.34	10.65	0.00
12	age + voles + TLCH + bc + TLCH x bc	9	-539.97	1098.35	10.66	0.00
13	age + voles + TLCH + bc + voles x TLCH + TLCH x bc	11	-537.96	1098.53	10.84	0.00
14	age + voles + TLCH + voles x TLCH	9	-540.33	1099.09	11.39	0.00
15	age + voles + TLCH + dist + NND + bc + voles x TLCH	12	-537.44	1099.62	11.93	0.00
16	voles + bc	6	-543.91	1100.02	12.32	0.00
17	age + voles + Hs_exp	7	-543.22	1100.71	13.02	0.00
18	age + voles + TLCH + voles x TLCH + voles x bc + TLCH x bc	13	-537.06	1100.97	13.28	0.00
19	age + voles + TLCH + age x TLCH	8	-542.52	1101.37	13.68	0.00
20	age + haem + voles + TLCH +	8	-542.63	1101.60	13.91	0.00
21	age + voles + TLCH + voles x bc + TLCH x bc	11	-539.52	1101.65	13.96	0.00
22	age + voles + TLCH + age x voles	9	-541.97	1102.37	14.67	0.00
23	age + voles + TLCH + dist + NND + bc + voles x bc	12	-539.26	1103.26	15.57	0.00
24	age + bc + voles x bc	8	-543.54	1103.42	15.73	0.00
25	voles + TLCH + bc	7	-544.60	1103.45	15.76	0.00
26	age + haem + voles + TLCH + voles x TLCH	10	-541.50	1103.51	15.81	0.00
27	age + voles + TLCH + age x TLCH + voles x TLCH	10	-541.67	1103.85	16.16	0.00
28	age + voles + TLCH + age x voles + voles x TLCH	11	-540.67	1103.96	16.27	0.00
29	age + voles + TLCH + dist + NND + bc + TLCH x bc	11	-540.89	1104.40	16.71	0.00
30	age + voles + TLCH + Hs_exp	8	-544.11	1104.56	16.86	0.00
31	age + voles + TLCH + dist + NND	9	-543.26	1104.94	17.25	0.00
32	age + voles + TLCH + dist + NND + bc + voles x TLCH + voles x bc	14	-538.06	1105.11	17.41	0.00
33	voles + TLCH + bc + voles x TLCH	9	-543.34	1105.11	17.42	0.00

Model nb	Candidate models response variable: lay date	df	LogLik	AICc	ΔAICc	ωi
34	age + voles + TLCH + dist + NND + bc + voles x TLCH + TLCH x bc	13	-539.26	1105.38	17.69	0.00
35	age + voles + TLCH + Hs_exp + voles x TLCH	10	-542.74	1105.99	18.29	0.00
36	voles	5	-547.95	1106.04	18.35	0.00
37	age + voles + TLCH + age x voles + age x TLCH	10	-542.78	1106.08	18.38	0.00
38	age + haem + voles + haem x voles	9	-543.86	1106.15	18.45	0.00
39	age + haem + voles + TLCH + haem x TLCH	9	-544.33	1107.08	19.39	0.00
40	voles + TLCH + bc + voles x bc	9	-544.33	1107.09	19.39	0.00
41	age + voles + TLCH + dist + NND + voles x TLCH	11	-542.32	1107.26	19.56	0.00
42	age + voles + TLCH + dist + NND + voles x bc + TLCH x bc	13	-540.54	1107.94	20.25	0.00
43	age + voles + TLCH + dist + NND + voles x TLCH + voles x bc + TLCH x bc	15	-538.41	1107.95	20.26	0.00
44	voles + dist	6	-548.10	1108.40	20.70	0.00
45	age + voles + TLCH + age x voles + age x TLCH + voles x TLCH	12	-541.99	1108.72	21.02	0.00
46	voles + TLCH + bc + voles x TLCH + voles x bc	11	-543.11	1108.84	21.14	0.00
47	age + voles + TLCH + dist + NND + age x TLCH	10	-544.20	1108.92	21.23	0.00
48	age + haem + voles + TLCH + haem x TLCH + voles x TLCH	11	-543.19	1109.01	21.31	0.00
49	age + haem + voles + TLCH + dist + NND	10	-544.41	1109.34	21.65	0.00
50	voles + TLCH	6	-548.65	1109.49	21.79	0.00
51	hame + voles	6	-548.90	1109.99	22.29	0.00
52	age + voles + TLCH + dist + NND + age x voles	11	-543.70	1110.02	22.32	0.00
53	age + haem + voles + TLCH + haem x voles	10	-544.76	1110.03	22.34	0.00
54	voles + TLCH + dist + NND + bc	9	-545.88	1110.19	22.49	0.00
55	voles + TLCH + bc + TLCH x bc	8	-547.05	1110.44	22.75	0.00
56	voles + NND	6	-549.37	1110.94	23.25	0.00
57	age + voles + TLCH + Hs_exp + TLCH x Hs_exp	9	-546.31	1111.04	23.35	0.00
58	voles + TLCH + voles x TLCH	8	-547.64	1111.61	23.92	0.00
59	age + haem + voles + TLCH + haem x voles + voles x TLCH	12	-543.47	1111.68	23.98	0.00
60	voles + TLCH + bc + voles x TLCH + TLCH x bc	10	-545.68	1111.87	24.18	0.00
61	age + voles + TLCH + Hs_exp + dist + NND	10	-545.71	1111.93	24.24	0.00
62	age + haem + voles + TLCH + dist + NND + voles x TLCH	12	-543.62	1111.97	24.27	0.00
63	age + voles + TLCH + dist + NND + age x TLCH + voles x TLCH	12	-543.67	1112.07	24.38	0.00
64	age + voles + Hs_exp + voles x Hs_exp	9	-547.00	1112.41	24.72	0.00
65	age + voles + TLCH + dist + NND + age x voles + voles x TLCH	13	-542.79	1112.44	24.75	0.00
66	voles + TLCH + dist + NND + bc + voles x TLCH	11	-545.02	1112.66	24.97	0.00
67	age + voles + TLCH + Hs_exp + voles x TLCH + TLCH x Hs_exp	11	-545.02	1112.66	24.97	0.00
68	voles + TLCH + voles x bc + TLCH x bc	10	-546.13	1112.77	25.08	0.00
69	voles + Hs_exp	6	-550.35	1112.90	25.20	0.00
70	haem + voles + TLCH	7	-549.57	1113.40	25.71	0.00
71	voles + TLCH + voles x TLCH + voles x bc + TLCH x bc	12	-544.39	1113.52	25.83	0.00
72	age + voles + TLCH + dist + NND + age x voles + age x TLCH	12	-544.60	1113.93	26.24	0.00
73	age + voles + TLCH + Hs_exp + dist + NND + voles x TLCH	12	-544.73	1114.20	26.51	0.00
74	age + voles + TLCH + dist + NND + bc + voles x TLCH + TLCH x bc	13	-539.26	1105.38	17.69	0.00

Model nb	Candidate models response variable: lay date	df	LogLik	AICc	ΔAICc	ωi
75	voles + TLCH + dist + NND + bc + voles x bc	11	-545.80	1114.21	26.52	0.00
76	age + voles + TLCH + haem x voles + haem x TLCH	11	-545.90	1114.42	26.72	0.00
77	age + haem + voles + TLCH + dist + NND + haem x TLCH	11	-546.11	1114.85	27.16	0.00
78	haem + voles + TLCH + voles x TLCH	9	-548.77	1115.96	28.27	0.00
79	age + voles + TLCH + haem x voles + haem x TLCH + voles x TLCH	13	-544.60	1116.07	28.37	0.00
80	age + voles + TLCH + Hs_exp + voles x Hs_exp	10	-547.86	1116.24	28.54	0.00
81	voles + TLCH + Hs_exp	7	-551.05	1116.36	28.67	0.00
82	voles + TLCH + dist + NND + bc + voles x TLCH + voles x bc	13	-544.89	1116.64	28.94	0.00
83	voles + TLCH + dist + NND + bc + TLCH x bc	10	-548.35	1117.22	29.52	0.00
84	age + voles + TLCH + dist + NND + age x voles + age x TLCH + voles x TLCH	14	-544.13	1117.25	29.56	0.00
85	voles + TLCH + dist + NND	8	-550.48	1117.29	29.59	0.00
86	age + haem + voles + TLCH + dist + NND + haem x voles	12	-546.36	1117.46	29.76	0.00
87	age + haem + voles + TLCH + dist + NND + haem x TLCH + voles x TLCH	13	-545.32	1117.50	29.80	0.00
88	age + voles + TLCH + Hs_exp + voles x TLCH + voles x Hs_exp	12	-546.51	1117.76	30.07	0.00
89	haem + voles + haem x voles	8	-550.96	1118.25	30.55	0.00
90	age + voles + TLCH + Hs_exp + dist + NND + TLCH x Hs_exp	11	-547.88	1118.37	30.68	0.00
91	voles + TLCH + Hs_exp + voles x TLCH	9	-550.08	1118.59	30.89	0.00
92	haem + voles + TLCH + haem x TLCH	8	-551.27	1118.87	31.17	0.00
93	voles + TLCH + dist + NND + bc + voles x TLCH + TLCH x bc	12	-547.33	1119.40	31.70	0.00
94	age + haem + voles + TLCH + dist + NND + haem x voles + voles x TLCH	14	-545.38	1119.75	32.05	0.00
95	voles + TLCH + dist + NND + voles x bc + TLCH x bc	12	-547.56	1119.85	32.16	0.00
96	voles + TLCH + dist + NND + voles x TLCH	10	-549.84	1120.19	32.50	0.00
97	age + voles + TLCH + Hs_exp + dist + NND + voles x TLCH + TLCH x Hs_exp	13	-546.99	1120.85	33.15	0.00
98	voles + TLCH + dist + NND + voles x TLCH + voles x bc + TLCH x bc	14	-546.12	1121.23	33.54	0.00
99	haem + voles + TLCH + haem x TLCH + voles x TLCH	10	-550.46	1121.44	33.75	0.00
100	age + voles + TLCH + voles x Hs_exp + TLCH x Hs_exp	11	-549.47	1121.57	33.87	0.00
101	haem + voles + TLCH + dist + NND	9	-551.58	1121.58	33.89	0.00
102	haem + voles + TLCH + haem x voles	9	-551.65	1121.71	34.02	0.00
103	age + voles + TLCH + dist + NND + haem x voles + haem x TLCH	13	-547.51	1121.88	34.19	0.00
104	voles + TLCH + Hs_exp + TLCH x Hs_exp	8	-553.36	1123.05	35.36	0.00
105	age + voles + TLCH + voles x TLCH + voles x Hs_exp + TLCH x Hs_exp	13	-548.21	1123.28	35.59	0.00
106	age + voles + TLCH + Hs_exp + dist + NND + voles x Hs_exp	12	-549.49	1123.71	36.01	0.00
107	haem + voles + TLCH + haem x voles + voles x TLCH	11	-550.69	1124.00	36.30	0.00
108	voles + TLCH + Hs_exp + dist + NND	9	-552.88	1124.17	36.48	0.00
109	age + voles + TLCH + dist + NND + haem x voles + haem x TLCH + voles x TLCH	15	-546.52	1124.18	36.49	0.00
110	voles + Hs_exp + voles x Hs_exp	8	-554.07	1124.48	36.79	0.00
111	haem + voles + TLCH + dist + NND + voles x TLCH	11	-551.10	1124.83	37.13	0.00
112	voles + TLCH + Hs_exp + voles x TLCH + TLCH x Hs_exp	10	-552.45	1125.42	37.72	0.00
113	age + voles + TLCH + Hs_exp + dist + NND + voles x TLCH + voles x Hs_exp	14	-548.53	1126.06	38.37	0.00
114	voles + TLCH + haem x voles + haem x TLCH	10	-552.78	1126.08	38.39	0.00
115	voles + TLCH + dist + NND + bc + voles x bc	11	-545.80	1114.21	26.52	0.00

Model nb	Candidate models response variable: lay date	df	LogLik	AICc	ΔAICc	ωi
116	haem + voles + TLCH + dist + NND + haem x TLCH	10	-553.27	1127.05	39.36	0.00
117	voles + TLCH + Hs_exp + dist + NND + voles x TLCH	11	-552.29	1127.19	39.50	0.00
118	voles + TLCH + Hs_exp + voles x Hs_exp	9	-554.74	1127.91	40.21	0.00
119	voles + TLCH + haem x voles + haem x TLCH + voles x TLCH	12	-551.82	1128.38	40.68	0.00
120	age + voles + TLCH + dist + NND + voles x Hs_exp + TLCH x Hs_exp	13	-551.06	1128.97	41.28	0.00
121	haem + voles + TLCH + dist + NND + haem x voles	11	-553.53	1129.67	41.98	0.00
122	voles + TLCH + Hs_exp + voles x TLCH + voles x Hs_exp	11	-553.80	1130.23	42.53	0.00
123	haem + voles + TLCH + dist + NND + haem x TLCH + voles x TLCH	12	-552.80	1130.32	42.63	0.00
124	voles + TLCH + Hs_exp + dist + NND + TLCH x Hs_exp	10	-555.16	1130.83	43.14	0.00
125	age + voles + TLCH + dist + NND + voles x TLCH + voles x Hs_exp + TLCH x Hs_exp	15	-550.19	1131.53	43.83	0.00
126	haem + voles + TLCH + dist + NND + haem x voles + voles x TLCH	13	-552.86	1132.58	44.89	0.00
127	voles + TLCH + voles x Hs_exp + TLCH x Hs_exp	10	-556.42	1133.35	45.66	0.00
128	voles + TLCH + Hs_exp + dist + NND + voles x TLCH + TLCH x Hs_exp	12	-554.63	1134.00	46.31	0.00
129	voles + TLCH + dist + NND + haem x voles + haem x TLCH	12	-554.67	1134.07	46.37	0.00
130	voles + TLCH + Hs_exp + dist + NND + voles x Hs_exp	11	-556.59	1135.80	48.10	0.00
131	voles + TLCH + voles x TLCH + voles x Hs_exp + TLCH x Hs_exp	12	-555.55	1135.83	48.14	0.00
132	voles + TLCH + dist + NND + haem x voles + haem x TLCH + voles x TLCH	14	-554.00	1136.99	49.30	0.00
133	voles + TLCH + Hs_exp + dist + NND + voles x TLCH + voles x Hs_exp	13	-556.02	1138.90	51.20	0.00
134	voles + TLCH + dist + NND + voles x Hs_exp + TLCH x Hs_exp	12	-558.23	1141.19	53.50	0.00
135	voles + TLCH + dist + NND + voles x TLCH + voles x Hs_exp + TLCH x Hs_exp	14	-557.73	1144.46	56.77	0.00
136	TLCH	4	-571.93	1151.96	64.26	0.00
137	(Null)	3	-573.98	1154.02	66.32	0.00

c) Complete model list following AICc ranking and model weights (ωi) for nestling survival (ratio of eggs that fledged successfully) in Eurasian kestrels *Falco tinnunculus*. Lay date (ld, centred to the mean of the study year), breeding experience (age), individual quality [either body condition (bc), *Haemoproteus* sp. infection risk (haem), individual genetic heterozygosity (Hs_exp)], territory land cover heterogeneity (Simpson's index, TLCH) and vole abundance in spring (voles) were fitted as fixed effects.. Additional co-variates were the distance to the closest forest edge (dist) and nearest neighbour distance (NND), both log-transformed. Nest box ID was used as random term. Model used for model averaging (ΔAICc < 4) in bold.

Model nb	Candidate models response variable: nestling survival	df	LogLik	AICc	ΔAICc	ωi
**1**	**age + voles + TLCH + ld + age x voles + age x TLCH + voles x TLCH**	**12**	**-509.00**	**1042.75**	**0.00**	**0.18**
**2**	**age + voles + TLCH + ld + age x voles + voles x TLCH**	**11**	**-510.14**	**1042.92**	**0.17**	**0.17**
**3**	**voles + TLCH + ld + voles x TLCH**	**8**	**-514.57**	**1045.48**	**2.72**	**0.05**
**4**	**age + voles + TLCH + ld + voles x TLCH**	**9**	**-513.58**	**1045.59**	**2.83**	**0.04**
**5**	**age + voles + TLCH + ld + dist + NND + age x voles + age x TLCH + voles x TLCH**	**14**	**-508.60**	**1046.21**	**3.46**	**0.03**
**6**	**age + voles + TLCH + ld + age x TLCH + voles x TLCH**	**10**	**-512.88**	**1046.28**	**3.53**	**0.03**
**7**	**age + voles + TLCH + ld + dist + NND + age x voles + voles x TLCH**	**13**	**-509.71**	**1046.29**	**3.54**	**0.03**
8	age + voles + TLCH + ld + age x voles	9	-514.20	1046.83	4.08	0.02
9	voles + TLCH + ld + bc + voles x TLCH	9	-514.21	1046.86	4.10	0.02
10	age + voles + TLCH + ld + voles x TLCH + voles x bc + TLCH x bc	13	-510.03	1046.94	4.19	0.02
11	age + voles + TLCH + ld + bc + voles x TLCH	10	-513.24	1047.00	4.25	0.02
12	age + voles + TLCH + ld + bc + voles x TLCH + TLCH x bc	11	-512.29	1047.21	4.46	0.02
13	voles + TLCH + Hs_exp + ld + voles x TLCH	9	-514.50	1047.42	4.67	0.02
14	age + voles + TLCH + Hs_exp + ld + voles x TLCH	10	-513.47	1047.46	4.70	0.02
15	voles + TLCH + ld + bc + voles x TLCH + TLCH x bc	10	-513.47	1047.47	4.71	0.02
16	haem + voles + TLCH + ld + voles x TLCH	9	-514.53	1047.50	4.74	0.02
17	age + haem + voles + TLCH + ld + voles x TLCH	10	-513.53	1047.59	4.84	0.02
18	age + voles + TLCH + ld + bc + voles x TLCH + voles x bc	12	-511.57	1047.89	5.14	0.01
19	voles + TLCH + ld + voles x TLCH + voles x bc + TLCH x bc	12	-511.62	1047.99	5.24	0.01
20	voles + TLCH + ld + bc + voles x TLCH + voles x bc	11	-512.84	1048.31	5.56	0.01
21	age + voles + TLCH + ld + age x voles + age x TLCH	10	-513.95	1048.42	5.67	0.01
22	haem + voles + TLCH + ld + haem x TLCH + voles x TLCH	10	-514.04	1048.60	5.85	0.01
23	age + haem + voles + TLCH + ld + haem x TLCH + voles x TLCH	11	-513.02	1048.68	5.93	0.01
24	age + voles + TLCH + ld + voles x bc + TLCH x bc	11	-513.04	1048.72	5.96	0.01
25	age + voles + TLCH + ld + dist + NND + voles x TLCH	11	-513.08	1048.80	6.05	0.01
26	voles + TLCH + ld + dist + NND + voles x TLCH	10	-514.14	1048.81	6.05	0.01
27	voles + TLCH + Hs_exp + ld + voles x TLCH + TLCH x Hs_exp	10	-514.50	1049.52	6.77	0.01
28	age + voles + TLCH + ld + dist + NND + age x TLCH + voles x TLCH	12	-512.40	1049.55	6.79	0.01
29	age + voles + TLCH + Hs_exp + ld + voles x TLCH + TLCH x Hs_exp	11	-513.46	1049.55	6.80	0.01
30	age + voles + TLCH + ld	7	-517.67	1049.60	6.84	0.01
31	voles + TLCH + ld	6	-518.79	1049.77	7.02	0.01
32	age + voles + TLCH + ld + dist + NND + voles x TLCH + voles x bc + TLCH x bc	15	-509.31	1049.79	7.04	0.01

Model nb	Candidate models response variable: nestling survival	df	LogLik	AICc	ΔAICc	ωi
33	haem + voles + TLCH + ld + haem x voles + voles x TLCH	11	-513.63	1049.90	7.14	0.01
34	age + haem + voles + TLCH + ld + haem x voles + voles x TLCH	12	-512.62	1050.00	7.24	0.00
35	voles + TLCH + ld + dist + NND + bc + voles x TLCH	11	-513.70	1050.04	7.28	0.00
36	age + voles + TLCH + ld + dist + NND + bc + voles x TLCH + TLCH x bc	13	-511.58	1050.04	7.28	0.00
37	age + voles + TLCH + ld + dist + NND + bc + voles x TLCH	12	-512.65	1050.04	7.29	0.00
38	voles + TLCH + Hs_exp + ld + voles x TLCH + voles x Hs_exp	11	-513.71	1050.06	7.30	0.00
39	age + voles + TLCH + ld + bc + TLCH x bc	9	-515.82	1050.08	7.32	0.00
40	age + voles + TLCH + Hs_exp + ld + voles x TLCH + voles x Hs_exp	12	-512.69	1050.13	7.38	0.00
41	voles + TLCH + ld + voles x bc + TLCH x bc	10	-514.90	1050.32	7.57	0.00
42	age + voles + TLCH + ld + dist + NND + age x voles	11	-513.92	1050.47	7.72	0.00
43	voles + TLCH + ld + dist + NND + bc + voles x TLCH + TLCH x bc	12	-512.86	1050.48	7.73	0.00
44	age + voles + TLCH + Hs_exp + ld + dist + NND + voles x TLCH	12	-512.98	1050.70	7.95	0.00
45	voles + TLCH + ld + bc + TLCH x bc	8	-517.21	1050.77	8.02	0.00
46	age + haem + voles + TLCH + ld + dist + NND + voles x TLCH	12	-513.01	1050.77	8.02	0.00
47	voles + TLCH + Hs_exp + ld + dist + NND + voles x TLCH	11	-514.07	1050.78	8.03	0.00
48	haem + voles + TLCH + ld + dist + NND + voles x TLCH	11	-514.09	1050.81	8.06	0.00
49	age + voles + TLCH + ld + dist + NND + bc + voles x TLCH + voles x bc	14	-510.99	1050.99	8.23	0.00
50	age + voles + TLCH + ld + bc	8	-517.33	1051.01	8.26	0.00
51	voles + TLCH + ld + dist + NND + voles x TLCH + voles x bc + TLCH x bc	14	-511.03	1051.08	8.32	0.00
52	age + voles + TLCH + Hs_exp + ld	8	-517.40	1051.14	8.39	0.00
53	voles + TLCH + ld + bc	7	-518.44	1051.15	8.40	0.00
54	age + voles + TLCH + ld + bc + voles x bc	10	-515.33	1051.19	8.44	0.00
55	voles + TLCH + ld + haem x voles + haem x TLCH + voles x TLCH	12	-513.32	1051.40	8.65	0.00
56	age + voles + TLCH + ld + age x TLCH	8	-517.54	1051.42	8.67	0.00
57	voles + TLCH + Hs_exp + ld	7	-518.59	1051.45	8.70	0.00
58	age + voles + TLCH + ld + haem x voles + haem x TLCH + voles x TLCH	13	-512.31	1051.50	8.74	0.00
59	age + haem + voles + TLCH + ld	8	-517.60	1051.55	8.79	0.00
60	voles + TLCH + ld + dist + NND + bc + voles x TLCH + voles x bc	13	-512.35	1051.58	8.82	0.00
61	age + haem + voles + TLCH + ld + dist + NND + haem x TLCH + voles x TLCH	13	-512.38	1051.64	8.89	0.00
62	haem + voles + TLCH + ld + dist + NND + haem x TLCH + voles x TLCH	12	-513.48	1051.71	8.96	0.00
63	haem + voles + TLCH + ld	7	-518.74	1051.75	9.00	0.00
64	age + voles + TLCH + ld + dist + NND + voles x bc + TLCH x bc	13	-512.45	1051.78	9.02	0.00
65	voles + TLCH + ld + bc + voles x bc	9	-516.77	1051.98	9.22	0.00
66	age + voles + TLCH + ld + dist + NND + age x voles + age x TLCH	12	-513.69	1052.14	9.39	0.00
67	voles + TLCH + ld + voles x TLCH + voles x Hs_exp + TLCH x Hs_exp	12	-513.71	1052.17	9.42	0.00
68	age + voles + TLCH + ld + voles x TLCH + voles x Hs_exp + TLCH x Hs_exp	13	-512.68	1052.24	9.48	0.00
69	age + haem + voles + TLCH + ld + haem x TLCH	9	-516.98	1052.39	9.64	0.00
70	haem + voles + TLCH + ld + haem x TLCH	8	-518.11	1052.57	9.81	0.00
71	age + haem + voles + TLCH + ld + dist + NND + haem x voles + voles x TLCH	14	-511.83	1052.67	9.92	0.00
72	haem + voles + TLCH + ld + dist + NND + haem x voles + voles x TLCH	13	-512.93	1052.75	9.99	0.00
73	age + voles + TLCH + Hs_exp + ld + dist + NND + voles x TLCH + TLCH x Hs_exp	13	-512.97	1052.82	10.06	0.00

Model nb	Candidate models response variable: nestling survival	df	LogLik	AICc	ΔAICc	ωi
74	voles + TLCH + Hs_exp + ld + dist + NND + voles x TLCH + TLCH x Hs_exp	12	-514.07	1052.90	10.15	0.00
75	age + voles + TLCH + ld + dist + NND	9	-517.35	1053.12	10.37	0.00
76	age + voles + TLCH + ld + dist + NND + bc + TLCH x bc	11	-515.27	1053.17	10.41	0.00
77	age + voles + TLCH + Hs_exp + ld + TLCH x Hs_exp	9	-517.40	1053.23	10.48	0.00
78	age + haem + voles + TLCH + ld + haem x voles	10	-516.36	1053.25	10.49	0.00
79	voles + TLCH + ld + dist + NND	8	-518.52	1053.38	10.63	0.00
80	age + voles + TLCH + Hs_exp + ld + dist + NND + voles x TLCH + voles x Hs_exp	14	-512.19	1053.39	10.64	0.00
81	haem + voles + TLCH + ld + haem x voles	9	-517.50	1053.44	10.68	0.00
82	voles + TLCH + Hs_exp + ld + dist + NND + voles x TLCH + voles x Hs_exp	13	-513.28	1053.44	10.68	0.00
83	voles + TLCH + Hs_exp + ld + TLCH x Hs_exp	8	-518.58	1053.51	10.75	0.00
84	voles + TLCH + ld + dist + NND + voles x bc + TLCH x bc	12	-514.43	1053.61	10.85	0.00
85	age + voles + TLCH + ld + dist + NND + haem x voles + haem x TLCH + voles x TLCH	15	-511.43	1054.02	11.26	0.00
86	voles + TLCH + ld + dist + NND + bc + TLCH x bc	10	-516.75	1054.03	11.28	0.00
87	voles + TLCH + ld + dist + NND + haem x voles + haem x TLCH + voles x TLCH	14	-512.54	1054.11	11.35	0.00
88	age + voles + TLCH + Hs_exp + ld + voles x Hs_exp	10	-516.81	1054.14	11.39	0.00
89	age + voles + TLCH + ld + dist + NND + bc	10	-516.93	1054.38	11.63	0.00
90	voles + TLCH + Hs_exp + ld + voles x Hs_exp	9	-518.00	1054.44	11.68	0.00
91	age + voles + TLCH + ld + haem x voles + haem x TLCH	11	-515.94	1054.51	11.76	0.00
92	age + voles + TLCH + ld + dist + NND + bc + voles x bc	12	-514.92	1054.59	11.83	0.00
93	voles + TLCH + ld + dist + NND + bc	9	-518.10	1054.63	11.87	0.00
94	voles + TLCH + ld + haem x voles + haem x TLCH	10	-517.07	1054.67	11.92	0.00
95	age + voles + TLCH + Hs_exp + ld + dist + NND	10	-517.08	1054.70	11.94	0.00
96	age + voles + TLCH + ld + dist + NND + age x TLCH	10	-517.24	1055.00	12.25	0.00
97	age + haem + voles + TLCH + ld + dist + NND	10	-517.26	1055.05	12.30	0.00
98	voles + TLCH + Hs_exp + ld + dist + NND	9	-518.33	1055.09	12.34	0.00
99	haem + voles + TLCH + ld + dist + NND	9	-518.46	1055.35	12.60	0.00
100	voles + TLCH + ld + dist + NND + bc + voles x bc	11	-516.43	1055.50	12.75	0.00
101	age + voles + TLCH + ld + dist + NND + voles x TLCH + voles x Hs_exp + TLCH x Hs_exp	15	-512.17	1055.51	12.76	0.00
102	voles + TLCH + ld + dist + NND + voles x TLCH + voles x Hs_exp + TLCH x Hs_exp	14	-513.28	1055.57	12.81	0.00
103	age + haem + voles + TLCH + ld + dist + NND + haem x TLCH	11	-516.55	1055.73	12.97	0.00
104	haem + voles + TLCH + ld + dist + NND + haem x TLCH	10	-517.74	1056.01	13.26	0.00
105	age + voles + TLCH + ld + voles x Hs_exp + TLCH x Hs_exp	11	-516.80	1056.24	13.49	0.00
106	age + haem + voles + TLCH + ld + dist + NND + haem x voles	12	-515.76	1056.27	13.51	0.00
107	voles + TLCH + ld + voles x Hs_exp + TLCH x Hs_exp	10	-517.99	1056.51	13.75	0.00
108	haem + voles + TLCH + ld + dist + NND + haem x voles	11	-516.98	1056.60	13.84	0.00
109	age + voles + ld + age x voles	8	-520.14	1056.61	13.86	0.00
110	age + voles + TLCH + Hs_exp + ld + dist + NND + TLCH x Hs_exp	11	-517.08	1056.80	14.05	0.00
111	voles + TLCH + Hs_exp + ld + dist + NND + TLCH x Hs_exp	10	-518.32	1057.17	14.41	0.00
112	age + voles + TLCH + ld + dist + NND + haem x voles + haem x TLCH	13	-515.25	1057.39	14.63	0.00
113	voles + TLCH + ld + dist + NND + haem x voles + haem x TLCH	12	-516.47	1057.69	14.93	0.00
114	age + voles + TLCH + Hs_exp + ld + dist + NND + voles x Hs_exp	12	-516.47	1057.69	14.94	0.00

Model nb	Candidate models response variable: nestling survival	df	LogLik	AICc	ΔAICc	ωi
115	voles + TLCH + Hs_exp + ld + dist + NND + voles x Hs_exp	11	-517.72	1058.08	15.33	0.00
116	voles +ld	5	-524.24	1058.62	15.86	0.00
117	age + voles + ld	6	-523.44	1059.08	16.33	0.00
118	age + voles + TLCH + ld + dist + NND + voles x Hs_exp + TLCH x Hs_exp	13	-516.47	1059.82	17.07	0.00
119	age + voles + ld + bc + voles x bc	9	-520.80	1060.04	17.28	0.00
120	voles + ld + NND	6	-523.92	1060.04	17.29	0.00
121	voles + ld + bc	6	-523.92	1060.05	17.29	0.00
122	voles + TLCH + ld + dist + NND + voles x Hs_exp + TLCH x Hs_exp	12	-517.71	1060.17	17.42	0.00
123	voles + Hs_exp + ld	6	-524.01	1060.22	17.46	0.00
124	voles + ld + bc + voles x bc	8	-521.95	1060.25	17.49	0.00
125	voles + ld + dist	6	-524.16	1060.52	17.77	0.00
126	age + voles + ld + bc	7	-523.13	1060.53	17.77	0.00
127	age + voles + Hs_exp + ld	7	-523.14	1060.55	17.80	0.00
128	haem + voles + ld	6	-524.21	1060.63	17.87	0.00
129	age + haem + voles + ld	7	-523.40	1061.08	18.32	0.00
130	haem + voles + ld + haem x voles	8	-523.29	1062.93	20.18	0.00
131	age + haem + voles + ld + haem x voles	9	-522.51	1063.45	20.69	0.00
132	voles +Hs_exp + ld + voles x Hs_exp	8	-523.66	1063.67	20.91	0.00
133	age + voles + Hs_exp + ld + voles x Hs_exp	9	-522.79	1064.01	21.26	0.00
134	voles	4	-531.05	1070.20	27.45	0.00
135	TLCH +ld	4	-534.04	1076.18	33.43	0.00
136	ld	3	-537.32	1080.69	37.94	0.00
137	ld + NND	4	-537.27	1082.63	39.87	0.00
138	ld + dist	4	-537.31	1082.71	39.96	0.00
139	(Null)	2	-546.13	1096.30	53.54	0.00

S8 Figure: Spatial distribution of 'individual quality' indices quantified in male (left panel, blue) and female (right panel, red) kestrels in the study area: (a) individual genetic heterozygosity (Hs_exp); (b) blood parasite infection; and (c) body condition index. Note variables were scaled and centred and are indicated in min, 1st quartile, median, 3rd quartile and max values in the figure legend.

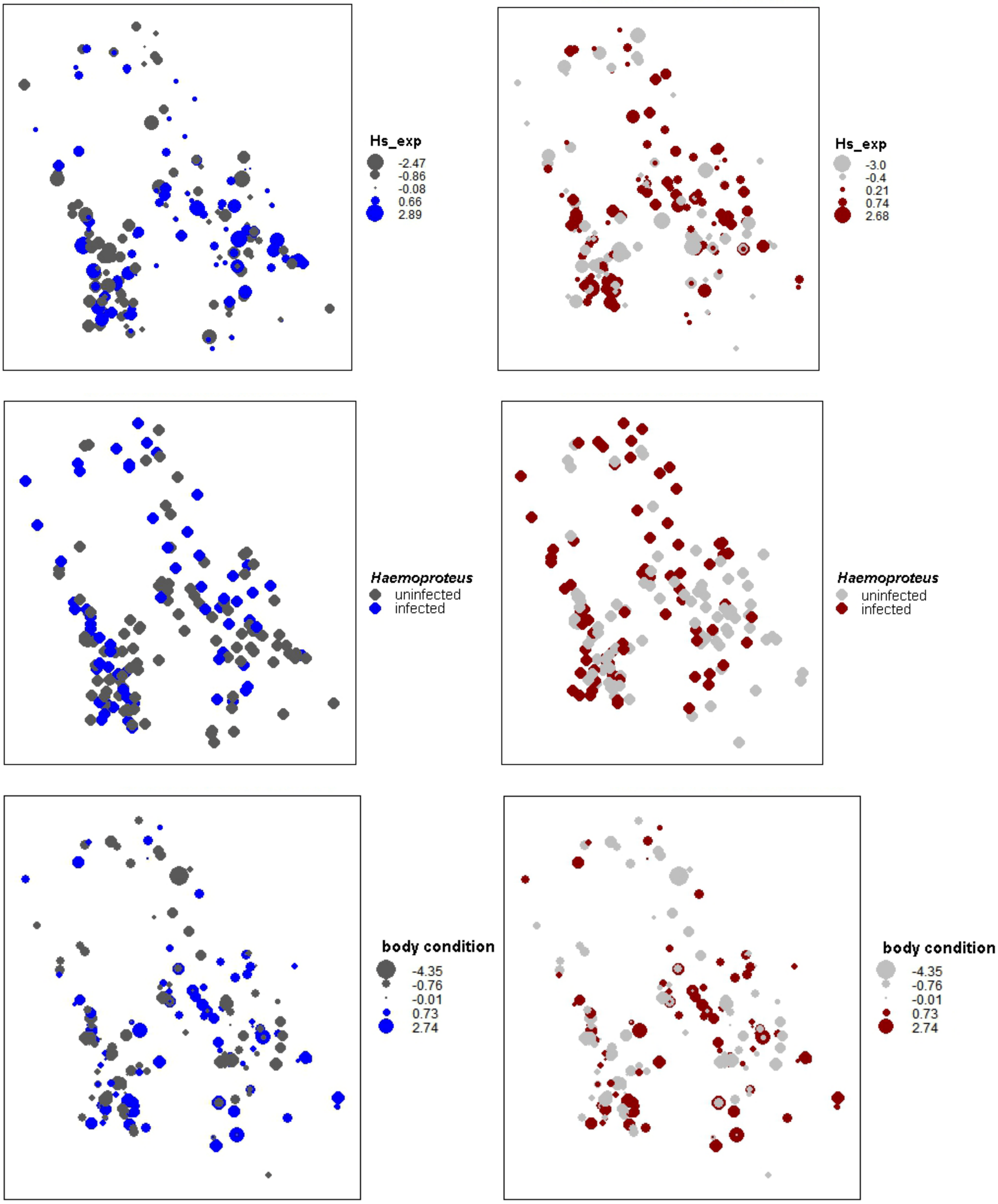

S9 Figure: Least square means post-doc contrasts of the interaction term age of the breeding adult, and the vole cycle, influencing nestling survival in Eurasian kestrels *Falco tinnunculus.*

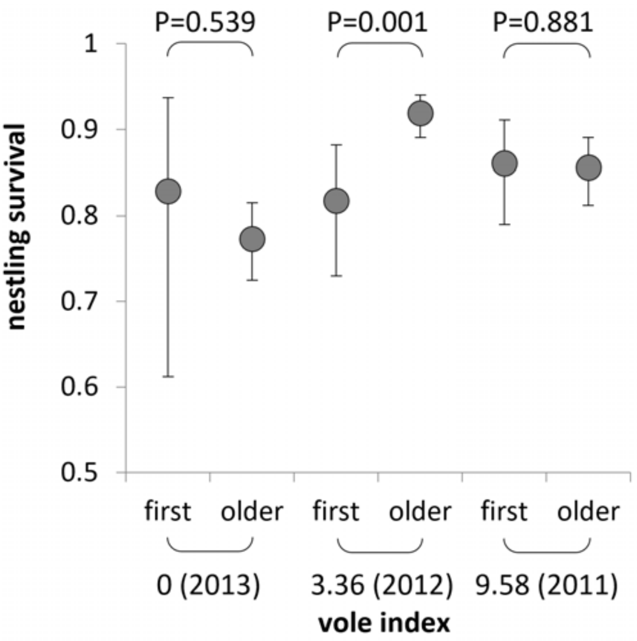

References supporting information:

Achard, S. 2012. brainwaver: Basic wavelet analysis of multivariate time series with a visualisation and parametrisation using graph theory. R package version 1.6.

Adamack, A. T. and Gruber, B. 2014. PopGenReport: simplifying basic population genetic analyses in R. - Methods in Ecology and Evolution 5: 384-387.

Aljanabi, S. M. and Martinez, I. 1997. Universal and rapid salt-extraction of high quality genomic DNA for PCR-based techniques. - Nucleic Acids Res. 25: 4692-4693.

Belkhir, K., et al. 2004. GENETIX 4.05, logiciel sous Windows TM pour la génétique des populations. Laboratoire Génome, Populations, Interactions, CNRS UMR 5000. - Université de Montpellier II, Montpellier (France)

Bensch, S., et al. 2009. MalAvi: a public database of malaria parasites and related haemosporidians in avian hosts based on mitochondrial cytochrome b lineages. - Molecular Ecology Resources 9: 1353-1358.

Chapman, J. R., et al. 2009. A quantitative review of heterozygosity–fitness correlations in animal populations. - Mol. Ecol. 18: 2746-2765.

Coulon, A., 2010. Genhet: An easy-to-use R function to estimate individual heterozygosity. – Mol. Ecol. Res. 10:167-169.

Hellgren, O., et al. 2004. A new PCR assay for simultaneous studies of *Leucocytozoon*, *Plasmodium*, and *Haemoproteus* from Avian Blood. - J. Parasitol. 90: 797-802.

Kalinowski, S. T., et al. 2007. Revising how the computer program cervus accommodates genotyping error increases success in paternity assignment. - Mol. Ecol. 16: 1099-1106.

Korpimäki, E. and Hakkarainen, H. 2012. The boreal owl: Ecology, behaviour and conservation of a forest-dwelling predator. - Cambridge University Press.

Matschiner, M. and Salzburger, W. 2009. TANDEM: integrating automated allele binning into genetics and genomics workflows. - Bioinformatics 25: 1982-1983.

Nesje, M., et al. 2000. Genetic relationships in the peregrine falcon (*Falco peregrinus*) analysed by microsatellite DNA markers. - Mol. Ecol. 9: 53-60.

Ortego, J., et al. 2007. Novel highly polymorphic loci and cross-amplified microsatellites for the lesser kestrel *Falco naumanni*. - Ardeola 54: 101-108.

Padilla, J., et al. 2009. Isolation and characterization of polymorphic microsatellite markers in lesser kestrel (*Falco naumanni*) and cross-amplification in common kestrel (*Falco tinnunculus*). - Conserv. Genet. 10: 1357-1360.

Raymond, M. and Rousset, F. 1995. GENEPOP (Version 1.2): Population Genetics Software for Exact Tests and Ecumenicism. - J. Hered. 86: 248-249.

Rousset, F. 2008. genepop’007: a complete re-implementation of the genepop software for Windows and Linux. - Molecular Ecology Resources 8: 103-106.

Valkiūnas, G., et al. 2014. Molecular characterization of five widespread avian haemosporidian parasites (Haemosporida), with perspectives on the PCR-based detection of haemosporidians in wildlife. - Parasitol. Res. 113: 2251-2263.

Van Oosterhout, C., et al. 2004. micro-checker: software for identifying and correcting genotyping errors in microsatellite data. - Mol. Ecol. Notes 4: 535-538.
